# A Comparative Assessment of the Risks of Introduction and Spread of Foot-and-Mouth Disease among Different Pig Sectors in Australia

**DOI:** 10.3389/fvets.2016.00085

**Published:** 2016-09-22

**Authors:** Marta Hernández-Jover, Nicole Schembri, Patricia K. Holyoake, Jenny-Ann L. M. L. Toribio, Peter Anthony Julian Martin

**Affiliations:** ^1^The University of Sydney, Farm Animal and Veterinary Public Health, Camden, NSW, Australia; ^2^Graham Centre for Agricultural Innovation, School of Animal and Veterinary Sciences, Charles Sturt University, Wagga, NSW, Australia; ^3^Department of Primary Industries Victoria, Epsom, VIC, Australia; ^4^Department of Agriculture and Food Western Australia, Bunbury, WA, Australia

**Keywords:** biosecurity, surveillance, emergency animal disease management, risk assessment, foot-and-mouth disease

## Abstract

Small-scale pig producers are believed to pose higher biosecurity risks for the introduction and spread of exotic diseases than commercial pig producers. However, the magnitude of these risks is poorly understood. This study is a comparative assessment of the risk of introduction and spread of foot-and-mouth disease (FMD) through different sectors of the pig industry: (1) large-scale pig producers; (2) small-scale producers (<100 sows) selling at saleyards and abattoirs; and (3) small-scale producers selling through informal means. An exposure and consequence assessments were conducted using the World Organization for Animal Health methodology for risk analysis, assuming FMD virus was introduced into Australia through illegal importation of infected meat. A quantitative assessment, using scenario trees and Monte Carlo stochastic simulation, was used to calculate the probabilities of exposure and spread. Input data for these assessments were obtained from a series of data gathering exercises among pig producers, industry statistics, and literature. Findings of this study suggest there is an *Extremely low* probability of exposure (8.69 × 10^−6^ to 3.81 × 10^−5^) for the three sectors of the pig industry, with exposure through direct swill feeding being 10–100 times more likely to occur than through contact with infected feral pigs. Spread of FMD from the index farm is most likely to occur through movement of contaminated fomites, pigs, and ruminants. The virus is more likely to spread from small-scale piggeries selling at saleyards and abattoirs than from other piggeries. The most influential factors on the spread of FMD from the index farm is the ability of the farmer to detect FMD, the probability of FMD spread through contaminated fomites and the presence of ruminants on the farm. Although small-scale producers selling informally move animals less frequently and do not use external staff, movement of pigs to non-commercial pathways could jeopardize animal traceability in the event of a disease outbreak. This study suggests that producers’ awareness on and engagement with legislative and industry requirements in relation to biosecurity and emergency animal disease management needs to be improved. Results from this study could be used by decision-makers to prioritize resource allocation for improving animal biosecurity in the pig industry.

## Introduction

Small landholders are commonly thought to pose biosecurity risks to mainstream livestock production, although the magnitude and significance of these risks have not been previously evaluated. Practices of small-scale pig producers are believed to be associated with a higher risk of introduction and spread of exotic diseases than those of larger producers (1–5). Previous research suggests that small-scale pig producers selling at livestock markets (saleyards) had poor on-farm biosecurity practices, poor disease knowledge and understanding of swill feeding, and limited veterinary contact (6–11). Similar concerns in relation to biosecurity and animal disease management were reported in a qualitative study among small-scale pig producers selling through informal means in Australia conducted in 2009, which provided an insight into the implementation of and attitudes toward biosecurity among this sector of the industry (12).

Characteristics of Australia, such as geographical isolation and quarantine procedures, provide the country with a privileged disease free status for major livestock diseases, like foot-and-mouth disease (FMD). However, the potential for the introduction of exotic diseases still exists. Illegal introduction of meat products by incoming passengers from infected countries is the highest risk source of entry of FMD into Australia (13–15). Between 1997 and 2000, there was a 29% increase in declared and detected animal products brought by passengers entering Australia from FMD-infected countries and for the highest risk group of countries the increase was 43% (14). Pigs are highly susceptible to FMD and once infected excrete high concentrations of the virus in aerosol form, being considered a major amplifying host for this disease (16). Feeding of infected meat scraps has been identified as one of the major pathways of introducing FMD into a free country (13, 15, 17). The source of a FMD outbreak in South Africa in 2000 was meat scraps from a ship’s garbage being fed to pigs (18) and during the 2001 FMD outbreak in the United Kingdom, a small-scale pig farm where unprocessed pig swill was fed to pigs, was considered the source case of the virus introduction (19). Similarly than for FMD, illegal introduction of meat products and subsequent swill feeding has also been suggested as the cause of outbreaks of classical swine fever (CSF) in the European Union in the 2000s and the introduction of African swine fever (ASF) in Eastern Europe in 2007 (20, 21). Previous studies have investigated the risk of introduction of and subsequent exposure to emergency animal diseases, such as FMD, CSF, and ASF, from the illegal importation of meat products (20, 22, 23). Hartnett et al. (22) quantified the risk of FMD introduction and exposure in Great Britain and Costard et al. (20) assessed the risk of ASF introduction and exposure in Europe. In both studies, poor biosecurity practices, especially among backyard producers and the presence of feral pigs were identified as highly influential on the probability of exposure of domestic pigs to these viruses.

The Productivity Commission (14) and Buetre et al. (24) assessed the impact of an FMD outbreak in Australia, considering a number of outbreaks of varying intensity. The most significant consequence of an FMD outbreak in Australia, independently of the location within the country, would be the immediate closure of the export market of livestock products to FMD-free countries, such as Japan and United States of America, which would remain for at least 3 months after eradication. The direct economic impacts of a FMD outbreak in Australia would be mainly due to the cost of control and eradication and a loss of revenue to affected livestock commodities from a decrease in export and domestic sales. The most recent assessment estimated a direct economic impact of $5.6 to $51.8 billion over a 10-year period, depending on the size of the outbreak. In addition, these financial effects would also have significant social impacts at an individual, household, and community levels, such as mental health issues and reduced welfare and well-being (24).

Spread of disease from the index farm will depend on on-farm biosecurity practices and animal movement patterns of pig enterprises. Understanding these practices among the different sectors of the pig industry is crucial to assess the risk of exotic disease introduction and spread posed by each of these sectors. This study conducts a comparative exposure and partial consequence assessment among different sectors of the pig industry in Australia. The aim of this study is to investigate how the FMD virus, which is assumed to be introduced into the country through illegal importation of contaminated meat, could exposure pigs at the index piggery and subsequently spread from this piggery. The sectors of the pig industry considered are: (1) large-scale or mainstream pig producers; (2) small-scale producers (<100 sows) selling through saleyards and abattoirs; and (3) small-scale producers selling through informal means. These assessments quantify the nature and magnitude of the biosecurity risks posed by each sector of the pig industry. This information could support decision-makers for the prioritization of resources allocation for improving biosecurity in the pig industry.

## Materials and Methods

### Exposure and Consequence Assessment Models

This comparative risk assessment follows the World Organization for Animal Health (OIE) methodology for risk analysis (25) and uses scenario tree models to represent the potential pathways of exposure and spread and subsequently calculate the corresponding probabilities of these occurring. Scenario trees provide an effective way of identifying pathways and information requirements and a framework for a quantitative analysis (26, 27).

An entry assessment as outlined by the OIE risk analysis methodology was not performed in this study as this assessment assumed that FMD had already been introduced into Australia. The assumption was that the virus was introduced through illegal importation of FMD-infected salted or cured meat and an estimated amount of introduced infected meat per year of 5 kg was used. The exposure assessment describes the potential pathways for pigs from the three different types of piggeries getting exposed to the FMD virus and estimates the probability of these pathways to occur. The partial consequence assessment describes the potential pathways of spread of FMD virus from the index farm and estimates the probability of this spread occurring. The assessment of the impacts of the resulting FMD outbreaks after virus introduction is not reported in this manuscript. The scenario trees were implemented in Microsoft Excel (PC/Windows XP, 2006) and probabilities were determined using Monte Carlo stochastic simulation modeling with the @RISK software (@Risk 6.0, Palisade Corporation, USA). The outcome probabilities for each pathway of exposure and spread were calculated as a product of all conditional probabilities describing the nodes of each specific pathway. The overall probability of exposure and spread for each type of piggery were obtained by adding the probabilities for each of the exposure and spread pathways, respectively, given these pathways are independent (27). Each simulation consisted of 50,000 iterations sampled using the Latin hypercube method with a fixed random seed of one.

#### Population Framework

Different definitions on small-scale pig producers can be found. The Australian pig body representative, Australian Pork Limited (APL), defines small landholder as those pig producers with less or equal than 50 sows and/or trading less or equal to 1000 pigs per year (28). Biosecurity Australia in their Import Risk Analysis for Pig Meat (13) classified backyard pig producers as those with less than 10 sows, small pig-producing enterprises those with between 10 and 99 sows, and commercial enterprises those with more than 99 sows. This classification was based on the assumption that management practices, such as feeding, husbandry, and motivation to keep pigs, where significantly different between these groups. Research into small-scale pig producers trading via saleyards in eastern Australia has shown no differences on on-farm practices among producers with 1–100 sows (8).

For the purpose of this assessment, small-scale pig producers are those with less than 100 sows with those piggeries with more than 100 sows being defined as commercial enterprises. Moreover, this assessment considers the differences on livestock trade patterns among small-scale pig producers as some livestock movements could pose higher risk for disease transmission. As a consequence, small-scale producers were subsequently classified into two groups; those selling through saleyards and abattoirs, and those selling mainly through informal means. Informal sales included internet, word-of-mouth, family and friends, and local businesses.

#### Data Sources

Data used to populate the exposure and partial consequence assessments were obtained from different data gathering exercises, published literature, and industry statistics. Below is a description of the data gathering exercises to collect information to populate the models used in these assessments.

##### Postal Questionnaire, Interviews, and Focus Groups with Pig Producers Selling through Saleyards in Eastern Australia

A three-part study involving pig producers at six saleyards situated in eastern Australia was conducted in a 12-month period starting at the end of 2006 (6). The first part of the study was the distribution of a postal questionnaire, which gathered basic data on the demographics and husbandry practices, among all producers who traded pigs at saleyards during the 2005 calendar year (*n* = 815). The second part of the study involved face-to-face interviews with producers (*n* = 106) who indicated their willingness to participate during the postal questionnaire, along with volunteers opportunistically recruited from the study saleyards. The interview collected detailed information on demographics, husbandry practices, nutrition, herd health, biosecurity practices, movement practices, animals identification systems, and communication networks (8, 9, 35, 42, 43). The final part of the study consisted in nine one-off focus group discussions, with 5–12 producers in each discussion, to investigate in depth attitudes and behaviors of producers toward diseases, disease reporting, traceability, and communication networks (6, 42–44). Focus group participants (*n* = 34) were recruited on a voluntary basis from face-to-face interviews and from advertisements placed at the saleyards and in stock agent newsletters. This study included mainly small-scale producers selling through saleyards although there were a small proportion of large-scale producers and small-scale producers selling through informal means.

##### Case Study Interviews and Questionnaires with Small-Scale Pig Producers Selling through Informal Means

To improve our understanding of practices of small-scale pig producers selling by informal means (internet, word-of-mouth, family and friends, and local businesses), a total of 13 small-scale (≤100 sows) pig producers using this marketing strategy were interviewed in New South Wales (12). This questionnaire, which was distributed using face-to-face interviews, gathered in-depth information on demographics and practices on husbandry, feeding, herd and health management, biosecurity, and pig movements. Producers were recruited at agricultural shows and through stage government databases. In addition, to collect supporting data in relation to practices of this sector of the pig industry, a shorter questionnaire covering similar topics was developed to be distributed by post among members of the Australian Pig Breeders Association (*n* = 29) and face-to-face among participants (*n* = 24) of pig industry field days.

#### Exposure Assessment

This assessment evaluates the probability of exposure of a pig from a piggery to FMD-infected meat that has been illegally introduced into the country. The assessment considers that the FMD-infected meat could end up in any household in the country, with or without pigs. Four different pathways have been identified as potential pathways of exposure of a pig at an index piggery for each of the three piggery types (small-scale piggery selling by informal means; small-scale piggery selling at saleyards and abattoirs; or large-scale piggery), depending on: (1) the type of household where the meat is destined to; (2) the proportion of waste discarded from this meat; (3) the involvement of feral pigs in the pathway; and (4) the probability of pig producers feeding swill to their pigs. These pathways of exposure are:

- *Exposure 1*: The FMD-infected meat ends up in a household without pigs and some of this meat is discarded as waste. This waste is then accessible to feral pigs and these pigs become infected with FMD. As the final step of the pathway, the infected feral pigs travel to the index piggery getting in contact with domestic pigs from this piggery.
- *Exposure 2*: The FMD-infected meat ends up in the index piggery and some of this meat is discarded as waste. This waste is then fed directly to the domestic pigs in the piggery.
- *Exposure 3*: The FMD-infected meat ends up in the index piggery and some of this meat is discarded as waste. This waste is then accessible to feral pigs around the piggery and these pigs become infected with FMD. The feral pigs get in contact with domestic pigs from the same piggery.
- *Exposure 4*: The FMD-infected meat ends up in a non-index piggery, and some of this meat is discarded as waste. This waste is then accessible to feral pigs and these pigs become infected with FMD. The infected feral pigs travel to the index piggery getting in contact with domestic pigs from this piggery.

**Figure 1:**
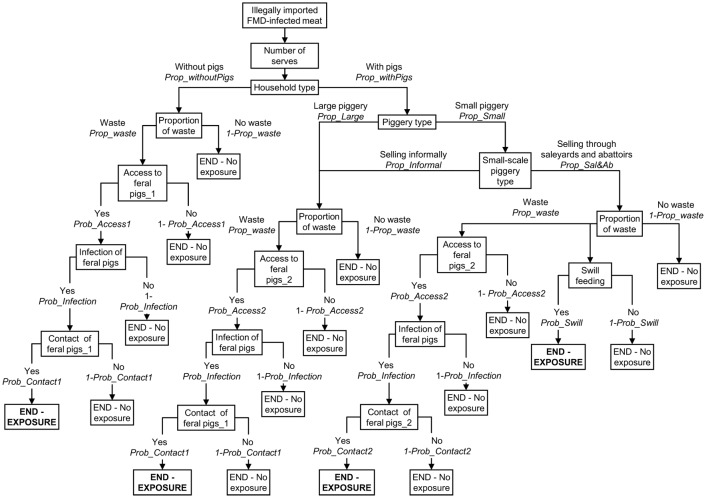
**Scenario tree representing the exposure pathways of a domestic pig from a small-scale piggery (<100 sows) selling through saleyards and abattoirs to foot-and-mouth disease (FMD) virus from FMD-infected meat illegally introduced into Australia**.

A scenario tree was developed to represent these four pathways of exposure and the same structure of the scenario tree was used for modeling the risk of FMD exposure among the three groups of pig producers considered in this study. Figure 1 represents the scenario tree considering a small-scale piggery selling through saleyards and abattoirs is the index piggery. Some of the parameter estimates and input values differed between the three groups of pig producers. Table 1 summarizes the nodes used for the exposure scenario tree and a detailed description of the nodes and input parameters used are provided in the online supplemental material.

**Table 1:** **Nodes, parameter estimates, and input values used for the exposure assessment estimating the probability of a piggery being exposed to FMD-infected meat illegally introduced into Australia through incoming passengers from overseas**.

	Name	Outcome	Parameter estimates	Input value	Data sources
1	Household type	Without pigs	Proportion of households with and without pigs in Australia (*Prop_withPigs; Prop_withoutPigs*)	Total households: Pert (8.7, 9.2, 9.6 M)Households with pigs: large piggery (>100 sows) + small piggery + growers Large piggery: 315 Growers: 524 Small piggery: Pert (1409, 1550, 620)	(8, 29, 30)
		With pigs			

2	Proportion of waste	Waste	Number of serves in 5 kg of meat	Single serve size of meat: average, 50 g (25–100 g), Pert (0.025, 0.05, 0.1)	(13, 31)
		No waste	Proportion of meat discarded as waste (*Prop_waste*)	Number of serves in 5 kg of meat: 5 kg/single serve size	
				*Prop_Waste*: Pert (0.01, 0.02, 0.05)	

3	Piggery type	Large piggery (> 100 sows)	Proportion of large and small-scale piggeries in Australia (*Prop_Small*; *Prop_Large*)	Large-scale piggeries: 839 (315 breeding, 524 contract growers) Small-scale piggeries: Pert (1071, 1178, 1232)	(8)
		Small piggery (<100 sows)			

4	Small-scale piggery type	Selling informally	Proportion of these two types of piggeries among small-scale piggeries (*Prop_Informal*; *Prop_Sal&Ab*)	Number small-scale piggeries: 589	Number of small-scale piggeries selling informally: Pert [Beta (38,553), +50%, +70%] (8, 12); Questionnaire with specific groups of pigs producers
		Selling through saleyards and abattoirs		Number of small-scale piggeries selling informally: Pert [Beta (38,553), +50%, +70%]	

5	Access of feral pigs to waste	Yes	Probability of waste from households without pigs getting in contact with feral pigs (*Prob_Access1*); probability of waste from piggeries getting in contact with feral pigs (*Prob_Access2*)	*Prop_Access1* = Cumul (Probability access and located, Proportion of households in different areas) Probability of access and located = Probability of waste being accessible [*High* (Uniform (0.7, 1)) in remote areas, *Moderate* (Uniform (0.3, 0.7)) in rural areas and *Very low* (Uniform (0.001, 0.05)) at large towns] × Probability of waste being located by the feral pigs *Very low* [Uniform (0.001, 0.05)] in remote areas, *Extremely low* [Uniform (0.000001, 0.001)] in rural areas and *Negligible* [Uniform (0, 0.000001)] at large towns Proportion of households: 3% remote, 11% rural, and 86% large towns *Prob_Access2* = Pert (*Prob_Access1*, +15%, +20%)	(13)
		No			

6	Infection of feral pigs	Yes	Probability of the feral pigs being infected once they are in contact with the FMD-contaminated waste (*Prob_Infection*)	*Prob_Infection* = Probability of the infected meat contains sufficient dose to cause infection of feral pigs *High* [Uniform (0.7, 1)] × Viability of the virus in the infected waste until the feral pig contacts with this waste *High* [Uniform (0.7, 1)]	(13, 15)
		No			

7	Contact of feral pigs with domestic pigs	Yes No	Probability feral pigs infected via waste from other households contact pigs from the index piggery (*Prob_Contact1*); probability feral pigs infected via waste originated in the index piggery contact pigs from the same piggery (*Prob_Contact2*)	*Prob_Contact2* = Proportion of producers reporting feral pigs around their property [Small-scale saleyard and abattoir, Beta (46, 127); small-scale selling informally, Beta (6, 17)]; large-scale, Pert (max–50%, max-20%, 23/149)	(8, 12); Questionnaire with specific groups of pigs producers
				*Prob_Contact1* = Pert (−50%, −20%, *Prob_Contact2*)	

8	Swill feeding	Yes No	Probability of swill feeding (*Prob_Swill*) among producers	Small-scale selling through saleyards and abattoirs: Pert (Most likely – 50%, 19/109, Most likely +20%)	(8, 9, 12); Questionnaire with specific groups of pigs producers
				Small-scale selling informally: Pert (Most likely – 50%, 5/22, Most likely +20%)	
				Large-scale: Pert (Most likely – 50%, 6/41, Most likely +20%)	

#### Consequence Assessment

Once the first pig from a piggery (small- or large scale) is exposed to the FMD virus, different potential outbreak scenarios could occur depending on different factors. This partial consequence assessment evaluates the potential outbreak scenarios and their corresponding probabilities occurring. The main factors considered in the consequence assessment are the ability of the farmer to detect the disease, the presence of ruminants on the farm and the movement of animals, fomites, and people from the index farm. In the event that the infection in the index farm is not detected, the virus could spread beyond this property. Six main outbreak scenarios have been identified:

- *Scenario 1*: This scenario represents no spread beyond the index farm, which could occur in different situations: (1) in a piggery with pigs only, when infection is detected and reported; (2) in a piggery with pigs and ruminants, when infection is detected in both species and reported; (3) in a piggery with pigs only, when infection is not detected, but there are no movement of pigs off the farm during the infective period either movements of contaminated fomites. In this last situation, the infection would die out before spread occurs. If ruminants are kept on the farm, these are very likely to become infected before FMD is detected in pigs and moved off the farm (see *Scenario 5*).
- *Scenario 2*: Infection is not detected (in pigs and ruminants) at the first exposed piggery and FMD virus is spread through movement of pigs off farm. This spread could be more or less significant and at local, regional, or national level, depending on the destination of the animals. Within *Scenario 2*, further scenarios were identified depending on the destination of the animals moving from the index farm when the infection is initially not detected by the farmer. These scenarios slightly differed between small and large-scale piggeries, and are described in Table 2.
- *Scenario 3*: Infection in the first exposed piggery is spread through movement of contaminated fomites. Spread through fomites can happen independently of farmer detection. If the farmer does not detect, spread of the virus to other properties through contaminated fomites is more likely than when detection occurs. However, if detection is delayed, spread through fomites can still happen. For this assessment, fomites were defined as mechanical vectors and included vehicles, equipment, and clothing.
- *Scenario 4*: Infection in the first exposed piggery is spread through movement of people carrying infective virus particles in the respiratory tract. As the previous scenario, spread through contaminated people is more likely to happen when the farmer does not detect the infection. When detection occurs but it is in late stages of the infection, spread through people could also be possible. Spread through people carrying the virus was considered separate to the spread through fomites as a person could be contaminated despite biosecurity measures being applied to avoid spread through fomites (e.g., disinfection of equipment).
- *Scenario 5*: Infection is not detected by the farmer (in pigs and ruminants) in the first exposed piggery and FMD virus is spread through movement of ruminants off farm. Even when infection is detected in pigs, movement of infected ruminants off the farm could occur before infection is detected in pigs. This spread could be more or less significant at a local, regional, or national level, depending on the destination of the animals. Information on the movement of ruminants off the farm, such as potential destinations and frequency of movements, was not collected during this assessment. However, information on the presence and number of ruminants kept on the farm was used to evaluate the likelihood of the spread through ruminant movement.
- *Scenario 6*: Infection in the first exposed piggery is spread through airborne transmission. Airborne transmission of FMD has been extensively investigated in the past (45, 46). It was not the objective of this assessment to assess the potential spread of FMD through airborne transmission as this could occur independently of the biosecurity practices of the piggery, evaluation of which is the main objective of this assessment.

**Table 2:** **A description of the potential scenarios of spread of FMD virus from an infected small-scale (<100 sows) or large-scale piggery in Australia, due to movement of pigs off farm, according to different destinations**.

Scenario	Piggery type	Description
a	Large/small scale	*Infected animals moving to another piggery (small-scale piggery for the small-scale scenario; large-scale piggery for the large-scale scenario) where the infection is detected and reported*: this scenario represents limited spread to a local pig community, depending on movement of other animals from the index farm until infection is detected at the second piggery, time of the detection at the second piggery, the presence of ruminants at this piggery and the movement of animals from this piggery before detection

b	Large/small scale	*Infected animals moving to another piggery (small-scale piggery for the small-scale scenario; large-scale piggery for the large-scale scenario) where the infection is not detected*: this scenario represents spread of the infection. The magnitude of this spread will depend on other animal movements from the index farm, the presence of ruminants at the second piggery, and the movement of animals from this piggery

c	Large scale	*Infected animals moving to a small-scale piggery where the infection is detected and reported*: this scenario is similar than *Scenario a*; however, the spread would be more limited as there would be less animals that could get infected at the second piggery

d	Large scale	*Infected animals moving to a small-scale piggery where the infection is not detected and infection is spread*: this scenario is similar than *Scenario b* although the spread would be more limited due to the lower number of animals that would be affected and could move from the second piggery

e	Small scale	*Infected animals are transferred to a person who keeps pigs as pets (private individual) and infection is detected and reported at this second location*: this scenario represents limited spread to a local pig community. Similar than *Scenario a*, the extent of the spread will depend on other animal movements from the first exposed piggery until infection is reported by the private individual, time of the detection and reporting at this second location, the presence of ruminants at this location and the movement of other animals from this location before detection

f	Small scale	*Infected animals are transferred to a person who keeps pigs as pets (private individual) and infection is not detected at this second location*: this scenario represents spread of the infection. The magnitude of this spread will depend on other animal movements from the first exposed piggery, the presence of ruminants at the second location and the movement of animals from this location. It is assumed that the extent of the spread of this scenario would be less significant than in *Scenario b* as a private individual keeping pigs as pets is less likely to move these or other animals off the farm

g	Small scale	*Infected animals moving to an agricultural show, where the infection is detected*: in this scenario, once the infection is detected, all movements to and from the show would be stopped. Spread to animals attending the agricultural show and also outside the show can occur depending on time of detection and movement of animals, fomites, and people off the show before detection

h	Small scale	*Infected animals moving to an agricultural show, where the infection is not detected*: spread of the infection would be more significant than any of the previous scenarios. Animals attending agricultural shows can travel from the same region, the same state, and also from other states in Australia. If infection is not detected at the show, all susceptible livestock could be infected, and infection could spread to any of the locations where animals are moved from the show. The spread could affect all susceptible livestock species at a local, regional, and national level, depending on destination of animals from the show and time of detection of the infection once infected animals move from this agricultural show

i	Small scale	*Infected animals moving to another property for home-kill*: independently of detection, animals will be killed so spread will be very limited, and its extent will depend on other animal movements from the first exposed piggery, the period of time until the infected animal is killed at the second property and the presence of ruminants in this property

j	Large/small scale	*Infected animals moving to a saleyard where infection is detected*: in this scenario, once infection is detected, all movements to and from the saleyard would stop. Spread would be limited to the first exposed piggery and animals attending the saleyard; however, spread could go beyond the animals attending at the saleyards if movement of animals and fomites from the saleyard occur before the detection of the infection. The extent of the spread of this scenario will also depend on other animal movements from the first exposed piggery until the infection is detected at the saleyard

k	Large/small scale	*Infected animals moving to a saleyard where infection is not detected*: spread of the infection could be significant at local and regional levels, although national spread could also occur as animals travel interstate to be sold at saleyards. Thus, the extent of the spread will depend on other animal movements from the first exposed piggery, movements of animals, and contaminated fomites from the saleyard and presence of ruminants at the saleyard

l	Large/Small-scale	*Infected animals moving to an abattoir where infection is detected*: once infection is detected at the domestic abattoir, all movements to and from the abattoir would be disrupted. This scenario represents a locally limited spread to the first exposed piggery and animals attending at the abattoir. Animal movement from the abattoir is uncommon; however, contaminated vehicles could spread the infection if they move from the abattoir before the infection is detected. The extent of the spread of this scenario will also depend on other animal movements from the first exposed piggery until the infection is detected at the abattoir

m	Large/small scale	*Infected animals moving to an abattoir where infection is not detected*: in this scenario, non-detected infected animals would be slaughtered and spread would most likely be limited to the local community, depending on other animal movements from the first exposed piggery and movement of infected animals and fomites from the abattoir

**Figure 2:**
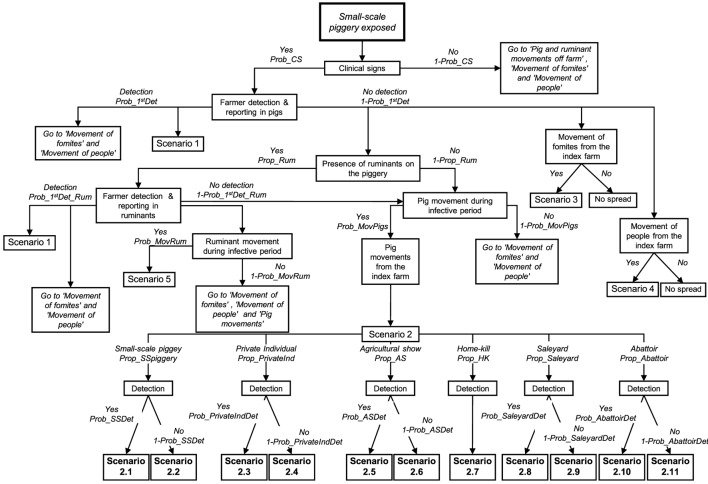
**Scenario tree used for a spread assessment investigating the potential outbreak scenarios of foot-and-mouth disease (FMD) from a small-scale piggery (<100 sows) in Australia**.

Two different consequence scenario trees were developed to represent the previously described potential outbreak scenarios for small and large-scale piggeries. The only difference in the structure of these scenario trees was the potential destinations where pigs from the index farm could go once moved off the farm. Figure 2 represents the consequence scenario tree used for modeling the probability of FMD spread from small-scale pig producers. Table 3 describes the nodes, input values and data sources used for the small-scale piggery consequence scenario tree. A description of the scenario tree used for the consequence assessment for large-scale piggeries is shown in Table 4. A detailed description of the nodes and input parameters used for both consequence assessments are provided in the online supplemental material.

**Table 3:** **Nodes, parameter estimates, and input values used for the partial consequence assessment estimating the probability of potential outbreak scenarios after a small-scale (<100 sows) piggery has been exposed to FMD-infected meat illegally introduced into Australia through incoming passengers from overseas**.

Node	Name	Outcome	Parameter estimates	Input value	Data sources
1	Clinical signs	Yes No	Probability that an FMD-infected animal would display clinical signs (*Prob_CS; Prob_CS_2*)	Incubation period: lognormal [5, 2.7, Truncate (1, 12)]; infective period: uniform (14, 30); Time to the onset of clinical signs = Incubation period – 2 days	(13, 15, 16)
				*Prob_CS* = (Infective period – Incubation period)/Infective period	
				*Prob_CS_2* = (Infective period – Time to the onset of clinical signs)/Infective period	

2	Farmer detection and reporting in pigs	Detection	Probability of the farmer from the index piggery detecting and reporting FMD in pigs for each type of piggery (*Prob_1^st^Det*)	*Prob_1^st^Det* = Probability of the farmer detection × Probability of the farmer reportingProbability of the farmer detection: Small-scale selling at saleyards and abattoirs: Pert (0.32, 0.4, 0.48) Small-scale selling informally: Pert (0.4, 0.5, 0.6) Probability of farmer reporting: Small-scale selling at saleyards and abattoirs: Pert (0.48, 0.6, 0.72) Small-scale selling informally: Pert (0.6, 0.75, 0.9)	(7, 8, 12); Questionnaire with specific groups of pigs producers
		No detection			

3	Presence of ruminants on the piggery	Yes	Proportion of pig farms keeping also ruminants (*Prop_Rum*)	Small-scale selling at saleyards and abattoirs: Beta (461,93)	As Node 2
		No		Small-scale selling informally: Beta (33, 6)	

4	Farmer detection & reporting in ruminants	Yes	Probability of the farmer from the first exposed piggery detecting and reporting the FMD infection in ruminants (*Prob_1^st^Det_Rum*)	*Prob_1^st^Det_Rum* = Probability of the farmer detection × Probability of the farmer reportingProbability of the farmer detection: Small-scale selling at saleyards and abattoirs: Pert (*Prob_1^st^Det*, +10%, +20%) Small-scale selling informally: Pert (*Prob_1^st^Det*, +10%, +20%) Probability of farmer reporting: Small-scale selling at saleyards and abattoirs: Pert (0.48, 0.6, 0.72) Small-scale selling informally: Pert (0.6, 0.75, 0.9)	(15)
		No			

5	Pig movement during infective period	Yes	Probability of pig movements during the infective period (*Prob_MovPigs*)	Small-scale selling at saleyards and abattoirs: Pert (−20%, 0.6, +20%)	As Node 2
		No		Small-scale selling informally: Pert (−20%, 0.3, +20%)	

6	Ruminant movement during infective period	Yes	Probability of ruminant movement during the infective period (*Prob_MovRum*)	Small-scale selling at saleyards and abattoirs: *Moderate* [Uniform (0.3, 0.7)]	As Node 2
		No		Small-scale selling informally: *Low* [Uniform (0.05, 0.3)]	

7	Pig movement from the index farm	Small scale	Proportion of movement of pigs to each of these destinations (*Prop_SSpiggery; Prop_PrivateInd; Prop_AS; Prop_HK; Prop_Saleyard; Prop_Abattoir*)	Beta (n + 1, s − n + 1) for each proportionMovements of small-scale selling at saleyards and abattoirs (*n* = 883): Small-scale piggery: 113 Private Individual: 26 Agricultural Show: 5 Home-kill: 192 Saleyard: 455 Abattoir: 82 Movements of small-scale selling informally (n = 57): Small-scale piggery: 32 Private Individual:10 Agricultural Show: 4 Home-kill: 0 Saleyard: 0 Abattoir: 1	As Node 2
		Piggery			
		Private individual			
		Agricultural show			
		Home-kill			
		Saleyard			
		Abattoir			

8	Ruminant movement from the index farm	Saleyard	Proportion of movement of ruminants to each of these destinations (*Prop_SaleyardRum; Prop_AbattoirRum; Prop_Contractor; Prop_IndProp; Prop_Export*)	*Not estimated*	(32–34)
		Abattoir			
		Contractor			
		Independent property			
		Export			

9	Detection at small-scale piggery	Detection	Probability that the farmer at the large-scale piggery receiving the infected pigs would detect infection (*Prob_SSDet*)	*Prob_SSDet* = Probability of the farmer detection × Probability of the farmer reporting Probability of the farmer detection: Pert (0.32, 0.4, 0.48) Probability of farmer reporting: Pert (0.48, 0.6, 0.72)	As Node 2
		No detection			

10	Detection at private individual	Detection	Probability that the farmer at the small-scale piggery receiving the infected pigs would detect infection (*Prob_PrivateIndDet*)	*Prob_PrivateIndDet* = Probability of the farmer detection × Probability of the farmer reporting Probability of the farmer detection: Pert (0.32, 0.4, 0.48) Probability of farmer reporting: Pert (0.48, 0.6, 0.72)	As Node 2
		No detection			

11	Detection at agricultural show	Detection	Probability of detection of FMD at agricultural shows (*Prob_ASDet*)	*Prob_ASDet* = SumProduct (Probability of detection at show type, Proportion of each show type) Proportion of show types depending on animal health responsible [Beta (n + 1, s − n + 1)]: Exhibitors only (17/59), Staff (25/59), Vet (17/59) Probability of detection at show type: Exhibitors only (*Prob_1^st^Det*), Staff [Pert (0.1, 0.3, 0.5)], Vet (1)	(35, 36)
		No detection			

12	Detection at home-kill	Detection	Probability of detection of a FMD in properties for home-kill (*Prob_HKDet*)	*Prob_HKDet* = Probability of the farmer detection × Probability of the farmer reporting Probability of the farmer detection: Pert (0.32, 0.4, 0.48) Probability of farmer reporting: Pert (0.48, 0.6, 0.72)	As Node 2
		No detection			

13	Detection at saleyards	Detection	Probability of detection of FMD at saleyards (*Prob_SaleyardDet*)	*Prob_SaleyardDet* = SumProduct (Probability of detection at saleyard type, Proportion of each saleyard type) Proportion of saleyard type: Domestic (10/13), Export (3/13) Probability of detection at saleyard type: Domestic (median, 0.475; 5–95%, 0.343–0.599), Export (0.474; 0.334–0.603)	(35, 37)
		No detection			

14	Detection at abattoirs	Detection	Probability of detection at pig domestic and export abattoirs (*Prob_AbattoirDet*)	*Prob_AbattoirDet* = SumProduct (Probability of detection at abattoir type, Proportion of each abattoir type) Proportion of saleyard type: Domestic (19/26), Export (7/26) Probability of detection at saleyard type: Domestic (0.430; 0.329–0.534), Export (0.861; 0.799–0.916)	(35, 37)
		No detection			

15	Movement of contaminated fomites from the index farm	Yes	Probability of FMD transmission through contaminated fomites (*Prob_Fomites*)	Small-scale selling at saleyards and abattoirs: *Moderate* [Uniform (0.3, 0.7)]	As Node 2; (15, 38, 39)
		No		Small-scale selling informally: *Low* [Uniform (0.05, 0.3)]	

16	Movement of contaminated people from the index farm^a^	Yes	Probability of transmission through movement of people carrying the virus in their respiratory tract (*Prob_People*)	Small-scale selling at saleyards and abattoirs: *Low* [Uniform (0.05, 0.3)]	As Node 2; (15, 40, 41)
		No		Small-scale selling informally: *Very low* [Uniform (0.001, 0.05)]	

*^a^Movement of people carrying FMD infective particles in the upper respiratory tract*.

**Table 4:** **Nodes, parameter estimates and input values used for the partial consequence assessment estimating the probability of potential outbreak scenarios after a large-scale (>100 sows) piggery has been exposed to FMD-infected meat illegally introduced into Australia through incoming passengers from overseas**.

Node	Name	Outcome	Parameter estimates	Input values	Data sources
1	Clinical signs	Yes No	Probability that an FMD-infected animal would display clinical signs (*Prob_CS; Prob_CS_2*)	Incubation period: lognormal [5, 2.7, Truncate (1, 12)]; infective period: uniform (14, 30); time to the onset of clinical signs = Incubation period – 2 days	(13, 15, 16)
				*Prob_CS* = (Infective period – Incubation period)/Infective period	
				*Prob_CS_2* = (Infective period – Time to the onset of clinical signs)/Infective period	

2	Farmer detection & reporting in pigs	Detection	Probability of the farmer from the index piggery detecting and reporting FMD in pigs (*Prob_1^st^Det*)	*Prob_1^st^Det* = Probability of the farmer detection × Probability of the farmer reporting Probability of the farmer detection: Pert (0.56, 0.7, 0.84) Probability of farmer reporting: Pert (0.64, 0.80, 0.96)	(7, 8, 12); Questionnaire with specific groups of pigs producers
		No detection			

3	Presence of ruminant on the piggery	Yes	Proportion of pig farms keeping also ruminants (*Prop_Rum*)	Beta (65, 24)	As Node 2
		No			

4	Farmer detection and reporting in ruminants	Yes	Probability of the farmer from the first exposed piggery detecting and reporting the FMD infection in ruminants (*Prob_1^st^Det_Rum*)	*Prob_1^st^Det_Rum* = Probability of the farmer detection × Probability of the farmer reporting Probability of the farmer detection: Pert (*Prob_1^st^Det*, +10%, +20%) Probability of farmer reporting: Pert (0.64, 0.80, 0.96)	(15)
		No			

5	Pig movement during infective period	Yes	Probability of pig movements during the infective period (*Prob_MovPigs*)	Pert (−10%, −5%, 1)	As Node 2
		No			

6	Ruminant movement during infective period	Yes	Probability of ruminant movement during the infective period (*Prob_MovRum*)	*High* [Uniform (0.7, 1)]	As Node 2
		No			

7	Pig movement from the index farm	Large-scale piggery	Proportion of movement of pigs to each of these destinations (*Prop_LCpiggery; Prop_SCpiggery; Prop_Saleyard; Prop_Abattoir*)	Large-scale piggery: Pert (−20%, 0.10, +20%)	As Node 2
		Small-scale piggery		Small-scale piggery: Pert (−20%, 0.10, +20%)	
		Saleyard		Saleyard: Pert (−20%, 0.15, +20%)	
		Abattoir		Abattoir: Pert (−20%, 0.65, +20%)	

8	Ruminant movement from the index farm	Saleyard	Proportion of movement of ruminants to each of these destinations (*Prop_SaleyardRum; Prop_AbattoirRum; Prop_Contractor; Prop_IndProp; Prop_Export)*	*Not estimated*	(32–34)
		Abattoir			
		Contractor			
		Independent property			
		Export			

9	Detection and reporting at large-scale piggery	Detection	Probability that the farmer at the large-scale piggery receiving the infected pigs would detect infection (*Prob_LSDet*)	*Prob_LSDet* = Probability of the farmer detection × Probability of the farmer reporting Probability of the farmer detection: Pert (0.56, 0.7, 0.84) Probability of farmer reporting: Pert (0.64, 0.80, 0.96)	As Node 2
		No detection			

10	Detection and reporting at small-scale piggery	Detection	Probability that the farmer at the small-scale piggery receiving the infected pigs would detect infection (*Prob_SSDet*)	*Prob_SSDet* = Probability of the farmer detection × Probability of the farmer reporting Probability of the farmer detection: Pert (0.32, 0.4, 0.48) Probability of farmer reporting: Pert (0.48, 0.6, 0.72)	As Node 2
		No detection			

11	Detection at saleyards	Detection	Probability of detection of FMD at saleyards (*Prob_SaleyardDet*)	*Prob_SaleyardDet* = SumProduct (Probability of detection at saleyard type, Proportion of each saleyard type) Proportion of saleyard type: Domestic (10/13), Export (3/13) Probability of detection at saleyard type: Domestic (median, 0.475; 5–95%, 0.343–0.599), Export (0.474; 0.334–0.603)	(35, 37)
		No detection			

12	Detection at abattoirs	Detection	Probability of detection at pig domestic and export abattoirs (*Prob_AbattoirDet*)	*Prob_AbattoirDet* = SumProduct (Probability of detection at abattoir type, Proportion of each abattoir type) Proportion of saleyard type: Domestic (19/26), Export (7/26) Probability of detection at saleyard type: Domestic (0.430; 0.329–0.534), Export (0.861; 0.799–0.916)	(35, 37)
		No detection			

13	Movement of contaminated fomites from the index farm	Yes	Probability of FMD transmission through contaminated fomites (*Prob_Fomites*)	*Moderate* [Uniform (0.3, 0.7)]	As Node 2; (15, 38, 39)
		No			

14	Movement of contaminated people from the index farm^a^	Yes	Probability of transmission through movement of people carrying the virus in their respiratory tract (*Prob_People*)	*Low* [Uniform (0.05, 0.3)]	As Node 2; (15, 40, 41)
		No			

*^a^Movement of people carrying FMD infective particles in the upper respiratory tract*.

### Sensitivity Analysis

The influence of some input parameters on the model outputs was investigated using the @Risk Advanced Sensitivity Analysis (@RISK 6.0, Palisade Corporation, USA). For the probability of exposure for each piggery type, the input parameters evaluated were the probability of pig producers swill feeding (*Prob_Swill)* and the amount of meat illegally introduced into Australia. For investigating which practices had the most influence on the probability of spread of the virus, the following spread scenarios were included in the sensitivity analysis: *Scenario 1* (no spread beyond the index farm), *Scenario 2* (spread through movement of pigs), *Scenario 3* (spread through contaminated fomites), and *Scenario 5* (spread through movement of ruminants). The input parameters investigated were the probability of the farmer detecting FMD in pigs (*Prob_1^st^Det*) and ruminants (*Prob_1^st^Det_Rum)*, the presence of ruminants on the farm (*Prop_Rum*), the probability of FMD transmission through contaminated fomites moving off the index farm (*Prob_Fomites*), and the probability of movement of pigs (*Prob_MovPigs*) and ruminants (*Prob_MovRum*) off the farm.

Probability input values were allowed to vary from 0 to 1 in tenths (0.1, 0.2, 0.3…) and the values used for the amount of meat illegally introduced into Australia were 10, 50, 100, and 200 kg. Each of the values for each input parameter was evaluated separately in a simulation of 5,000 iterations, while values for all other input variables were fixed to the base value.

## Results

### Exposure Assessment Results

The exposure assessment evaluated potential pathways of exposure of a pig from a piggery to FMD-infected meat illegally introduced into Australia and estimated the probability of these pathways to occur. Four potential exposure pathways were identified in this assessment, which were mainly dependent on the type of household in Australia where the contaminated meat was destined to and the characteristics of these households. Description of the exposure pathways, the likelihood of each of these pathways to occur and the overall likelihood of exposure for the three assessments (small-scale pig producers selling through informal means, small-scale pig producers selling at saleyards and abattoirs and large-scale pig producers) are presented in Table 5.

**Table 5:** **Quantitative (median, 5 and 95 percentiles) and qualitative estimates of the likelihood of exposure of a pig from a piggery to FMD-infected meat previously illegally introduced into Australia, according to potential pathways of exposure and piggery type**.

Exposure pathway	Description^a^	Quantitative and qualitative estimates^b^		
		Small-scale piggeries selling through informal means	Small-scale piggeries selling at saleyards and abattoirs	Large-scale piggeries
1	The FMD-infected meat gets to a household without pigs and some is discarded as waste – waste accessible to feral pigs, which become infected – Infected feral pigs get in contact with a pig from the exposure piggery	1.51 × 10^−7^	1.59 × 10^−7^	7.62 × 10^−8^
		(1.32 × 10^−8^ to 2.74 × 10^−4^)	(1.45 × 10^−8^ to 2.99 × 10^−4^)	(7.30 × 10^−9^ to 1.00 × 10^−4^)
		Negligible	Negligible	Negligible
2	The FMD-infected meat gets to the exposure piggery and some is discarded as waste – waste is directly fed to a pig from the same the piggery	7.80 × 10^−6^	6.65 × 10^−5^	3.46 × 10^−5^
		(3.82 × 10^−6^ to 1.66 × 10^−5^)	(3.29 × 10^−5^ to 1.33 × 10^−4^)	(1.65 × 10^−5^ to 7.14 × 10^−5^)
		Extremely low	Extremely low	Extremely low
3	The FMD-infected meat gets to the exposure piggery and some is discarded as waste – waste accessible to feral pigs, which become infected – infected feral pigs get in contact with a pig from the same piggery	4.00 × 10^−12^	4.23 × 10^−11^	1.19 × 10^−11^
		(3.72 × 10^−13^ to 6.91 × 10^−9^)	(3.94 × 10^−12^ to 7.63 × 10^−8^)	(1.10 × 10^−12^ to 1.59 × 10^−8^)
		Negligible	Negligible	Negligible
4	The FMD-infected meat gets to a non-exposure piggery, and some is discarded as waste – waste accessible to feral pigs, which become infected – infected feral pigs get in contact with a pig from the exposure piggery	4.98 × 10^−11^	2.43 × 10^−11^	1.64 × 10^−11^
		(4.16 × 10^−12^ to 8.80 × 10^−8^)	(2.23 × 10^−12^ to 4.54 × 10^−8^)	(1.54 × 10^−12^ to 2.22 × 10^−8^)
		Negligible	Negligible	Negligible

Overall	The FMD-infected meat gets in contact with pigs from the exposure piggery	8.69 × 10^−6^	7.26 × 10^−5^	3.81 × 10^−5^
		(4.03 × 10^−6^ to 2.83 × 10^−4^)	(3.36 × 10^−5^ to 3.98 × 10^−4^)	(1.77 × 10^−5^ to 1.40 × 10^−4^)
		Extremely low	Extremely low	Extremely low

*^a^Exposure piggery = small-scale pig producers selling through informal means, small-scale pig producers selling through saleyards and abattoirs or large-scale pig producers according to the assessment*.

*^b^Quantitative estimates are the output distribution of a simulation stochastic model with 50,000 iterations; qualitative estimates are based on the median and the likelihood ranges described at the Guidelines for Import Risk Analysis (DAFF, 2004)*.

Results indicate that the most likely pathway of exposure to FMD-infected meat illegally introduced into Australia among the three types of piggeries is through the direct feeding of the infected meat to the pigs (*Exposure 2*). The probability of this exposure pathway to occur is *extremely low* (qualitative descriptors based on Guidelines for Import Risk Analysis; DAFF, 2004), with one exposure estimated to occur for every 10,000–100,000 times FMD-infected meat is illegally introduced into the country, depending on the type of piggery. The lowest value was estimated for small-scale piggeries selling informally as shown in Table 5. Probabilities of other pathways were considered to be *negligible*. Exposure 1, which represents the exposure through feral pigs which have been infected from contaminated waste from a household without pigs, has a higher probability to occur than the other two pathways, given most households in Australia do not have pigs (99.9%) and the illegally introduced meat is more likely to be destined to these households than households with pigs. The overall probability of exposure was estimated 8.69 × 10^−6^, 7.26 × 10^−5^, and 3.81 × 10^−5^ (*extremely low*), for small-scale piggeries selling informally, those selling through saleyards and abattoirs and large-scale piggeries, respectively. The probabilities of exposure among small-scale piggeries selling through saleyards and abattoirs and large-scale piggeries are slightly higher than that for small-scale piggeries selling informally, due to the higher number of producers within the former categories, and the higher potential contact between feral and domestic pigs in these piggeries.

This assessment considered that households without and with pigs had even probability of illegally introducing meat into the country. When evaluating the probability of exposure of the pigs to the infected meat, once this meat is introduced to the piggery of concern, a *very low* probability (0.003–0.005) was obtained for all piggery types, given most of this meat will be consumed and only a small proportion will be waste that could be fed to the pigs.

### Consequence Assessment Results

Following the exposure of a pig from a piggery, six main scenarios have been identified depending on the ability of the farmer to detect FMD and the movement of animals, fomites, and people from the index farm. These scenarios are the same for the three groups of pig producers.

#### Assessment of the Risks of FMD Spread from Small-Scale Pig Producers

Table 6 shows a description of the potential outbreak scenarios and the likelihood of each of these scenarios to occur once FMD virus has been introduced into a small-scale piggery. The likelihood of five of the six main potential outbreak scenarios has been evaluated in this assessment. Among these scenarios and for small-scale producers selling through informal means, the most likely potential outbreak scenarios, with a similar likelihood of occurring, are *Scenario 1, Scenario 2, Scenario 3*, and *Scenario 5. Scenario 1*, representing no spread of FMD beyond the index farm, has a 0.193 (*Low*) probability to occur, as the ability of the farmer to detect FMD in pigs is estimated low. The probability of *Scenarios 2* and *5* to occur is estimated 0.137 and 0.100, respectively (*Low*). In these scenarios, the FMD-infected animals at the index farm are not detected and the virus spread from this farm through movement of pigs or ruminants. The extent of the spread and the impact of the consequences will depend on the destination of the animals moving off the farm. The likelihood of *Scenario 3* to occur is estimated similar, with a probability of 0.175 (*Low*). This scenario represents the spread of the FMD virus through the movement of contaminated fomites independently of the farmer detection. The virus could spread to another farm, saleyards, or abattoirs. The low frequency of movement of pigs and ruminants from this type of piggeries is the main reason why there is a low probability of spread through movement of animals (*Scenarios 2* and *5*) and fomites (*Scenario 3*).

**Table 6:** **Quantitative (median, 5 and 95 percentiles) and qualitative estimates of the likelihood of the potential outbreak scenarios for the introduction and spread of foot-and-mouth disease in small-scale piggeries in Australia**.

Outbreak Scenarios	Quantitative and qualitative estimate^a^		Description^b^
	Small-scale piggery selling through informal means	Small-scale piggery selling at saleyards and abattoirs	
1	0.193 (0.122–0.272), Low	0.092 (0.060–0.122), Low	Infection detected at the exposure farm: no FMD spread beyond the exposure farm
2	0.137 (0.107–0.171), Low	0.382 (0.325–0.445), Moderate	Infection not detected at the exposure farm: spread of FMD through movement of pigs off farm (any destination)
a	0.006 (0.003–0.012), Very low	0.014 (0.010–0.018), Very low	FMD spread to another small-scale piggery where infected pigs are detected
b	0.102 (0.074–0.132), Low	0.054 (0.042–0.067), Low	FMD spread to another small-scale piggery where infected pigs are not detected
e	0.011 (0.006–0.017), Very low	0.004 (0.003–0.006), Very low	FMD spread to a private individual (pigs kept as pets) where infected pigs are detected
f	0.031 (0.018–0.050), Very low	0.012 (0.008–0.017), Very low	FMD spread to a private individual (pigs kept as pets) where infected pigs are not detected
g	0.009 (0.004–0.018), Very low	0.002 (0.001–0.003), Very low	FMD spread to an agricultural show where infected pigs are detected
h	0.009 (0.004–0.019), Very low	0.002 (0.001–0.004), Very low	FMD spread to an agricultural show where infected pigs are not detected
i	0.035 (0.021–0.054), Very low	0.097 (0.080–0.117), Low	FMD spread to a property where pigs are killed for home consumption (home-kill)
j	0.001 (0.000–0.005), Very low	0.119 (0.090–0.153), Low	FMD spread to a saleyard (livestock market) where infected pigs are detected
k	0.001 (0.000–0.006), Very low	0.133 (0.099–0.168), Low	FMD spread to a saleyard (livestock market) where infected pigs are not detected
l	0.003 (0.000–0.008), Very low	0.020 (0.013–0.027), Very low	FMD spread to a domestic abattoir where infected pigs are detected
m	0.003 (0.001–0.010), Very low	0.026 (0.019–0.033), Very low	FMD spread to a domestic abattoir where infected pigs are not detected
3	0.175 (0.062–0.287), Low	0.499 (0.320–0.680), Moderate	Spread of FMD virus from the index farm through movement of contaminated fomites
4	0.026 (0.003–0.048), Very low	0.175 (0.062–0.287), Low	Spread of FMD virus from the index farm through movement of people carrying infective particles of virus in the respiratory tract
5	0.100 (0.037–0.183), Low	0.421 (0.270–0.600), Moderate	Infection not detected at the exposure farm: spread of FMD through movement of ruminants off farm (any destination)

*^a^Quantitative estimates are the output distribution of a simulation stochastic model with 50,000 iterations; qualitative estimates are based on the median and the likelihood ranges described at the Guidelines for Import Risk Analysis (DAFF, 2004)*.

*^b^Exposure piggery = small-scale pig producers selling through informal means or small-scale pig producers selling through saleyards and abattoirs according to the assessment*.

Within Scenario 2, Table 2 describes the potential outbreak scenarios through movement of pigs from a small-scale piggery selling informally according to the destination of the FMD-infected pigs. The most likely scenario is the spread of FMD virus to another small-scale piggery where the FMD-infected pigs are not detected (*Scenario b*), with a likelihood of occurring of 0.102 (*Low*). This scenario is more likely to occur than the rest of scenarios involving movement of pigs off the index farm as the main destination of pigs from a small-scale piggery selling through informal means is another small-scale piggery. The less likely scenarios are those involving spread to saleyards and abattoirs.

Among the five main potential outbreak scenarios from a small-scale piggery selling at saleyards and abattoirs, the most likely scenarios are *Scenario 2, Scenario 3*, and *Scenario 5*, all with a *Moderate* likelihood of occurring (Table 6). The likelihood of *Scenarios 2* and *3* for this group of producers was estimated higher than those obtained for small-scale piggeries selling through informal means, given movements of pigs from small-scale piggeries selling at saleyards and abattoirs were reported to be more frequent than those from small-scale piggeries selling informally and a lower proportion of these producers had boots and/or overalls for on-farm use only. Spread of FMD virus through movement of people carrying the virus in their respiratory tract (*Scenario 4)* was estimated to be 0.175 (*Low*) as the virus only survives for up to 28 h in the human respiratory tract, these farms do not usually employ external staff to work in the piggery, and over half of these farms have controlled entry of visitors and wash their hands after handling the pigs. However, this estimate is higher than the estimate for small-scale piggeries selling through informal means, as the latter group of producers have better on-farm practices, which could avoid the spread of the virus through contaminated people. *Scenario 1* (0.092, *Low*), representing detection of FMD at the index farm and no spread beyond this farm, has a similar estimate than for the previous group of producers. This scenario has the lowest probability of occurring as the probability of the farmer detecting and reporting at these piggeries was estimated lower than for the other groups of producers.

Regarding those scenarios involving movement of pigs off the index farm to different destinations, the most likely scenarios to occur in this group of pig producers are *Scenario j* (0.119, *Low*) and *k* (0.133, *Low*), representing movement of pigs to saleyards, where infection is or is not detected, respectively.

#### Assessment of the Risks of FMD Spread from Large-Scale Pig Producers

The six main outbreak scenarios are the same than those described for small-scale pig producers; however, scenarios involving movement of pigs off the index farm differed depending on the destination of these animals. Table 7 shows the potential outbreak scenarios and the likelihood of each of these scenarios to occur once FMD virus has been introduced into a large-scale piggery. If FMD is introduced into a large-scale piggery, the most likely scenarios to occur are *Scenario 1* and *Scenario 3*. *Scenario 1*, with a probability of 0.317 (*Moderate*), represents detection of FMD at the index farm and no spread beyond this farm. The likelihood of this scenario in large-scale piggeries is higher than that in both groups of small-scale piggeries, as the ability to detect disease among large-scale producers is estimated higher than that for small-scale producers and there is a lower proportion of large-scale producer with ruminants on the farm, limiting the potential spread off the farm through this species. The likelihood of *Scenario 3* to occur was estimated 0.499 (*Moderate*). This estimate is the same than the estimate for this scenario for small-scale piggeries selling at saleyards and abattoirs. Spread of FMD through movement of pigs (*Scenario 2*) when the infection has not been detected has a probability of occurring of 0.263 (*Low*). This probability is higher than that for the same scenario in small-scale piggeries selling through informal means, due to the more frequent movements of pigs, but lower than that estimated for small-scale piggeries selling through saleyards and abattoirs, as the producer in a large-scale piggeries is more likely to detect FMD than that in the small-scale piggery. Similarly, the likelihood of spread through movement of ruminants (*Scenario 5*; 0.218, *Low*) is lower than that in small-scale piggeries selling at saleyards and abattoirs but higher than that in small-scale piggeries selling informally.

**Table 7:** **Quantitative (median, 5 and 95 percentiles) and qualitative estimates of the likelihood of the potential outbreak scenarios for the introduction and spread of foot-and-mouth disease in large-scale piggeries (>100 sows) in Australia**.

Outbreak Scenarios	Quantitative and qualitative estimate^a^	Description
1	0.317 (0.199–0.444), Moderate	Infection detected at the exposure farm: no FMD spread beyond the exposure farm
2	0.263 (0.176–0.372), Low	Infection not detected at the exposure farm: spread of FMD through movement of animals off farm (any destination)
a	0.024 (0.018–0.032), Very low	FMD spread to another large-scale piggery where infected pigs are detected
b	0.023 (0.016–0.035), Very Low	FMD spread to another large-scale piggery where infected pigs are not detected
c	0.011 (0.008–0.014), Very low	FMD spread to a small-scale piggery where infected pigs are detected
d	0.044 (0.032–0.055), Very Low	FMD spread to a small-scale piggery where infected pigs are not detected
j	0.031 (0.021–0.042), Very low	FMD spread to a saleyard (livestock market) where infected pigs are detected
k	0.039 (0.028–0.059), Very low	FMD spread to a saleyard (livestock market) where infected pigs are not detected
l	0.157 (0.118–0.197), Low	FMD spread to an abattoir where infected pigs are detected
m	0.150 (0.105–0.235), Low	FMD spread to an abattoir where infected pigs are not detected
3	0.499 (0.320–0.680), Moderate	Spread of FMD virus from the index farm through movement of contaminated fomites
4	0.175 (0.062–0.287), Low	Spread of FMD virus from the index farm through movement of people carrying infective particles of virus in the respiratory tract
5	0.218 (0.0.116–0.334), Low	Infection not detected at the exposure farm: spread of FMD through movement of ruminants off farm (any destination)

*^a^Quantitative estimates are the output distribution of a simulation stochastic model with 50,000 iterations; Qualitative estimates are based on the median and the likelihood ranges described at the Guidelines for Import Risk Analysis (DAFF, 2004)*.

Among the scenarios of spread due to movement of pigs off the index farm (*Scenario 2*), the most likely scenarios were those where pigs were sent to the abattoir (*Scenario l* and *m*, approximately 0.150, *Low*), as this was the most common destination of pigs from large-scale piggeries.

### Sensitivity Analysis

The sensitivity of the probability of exposure and the spread scenarios to some of the input variables was evaluated in this assessment. The input variable with most influence on the probability of exposure for all exposure groups is the amount of FMD-infected meat illegally introduced into Australia. However, even when the model assumes that 200 kg of FMD-infected meat has been introduced into Australia, the probability that pigs would be exposed to FMD is *Very low* in large-scale and small-scale producers selling at saleyards and abattoirs and *Extremely low* in small-scale producers selling informally. When the probability of producers feeding swill to the pigs was increased to 0.9, there was a two- to sevenfold increase in the probability of exposure for all exposure groups; however, the probability was still considered *Extremely low*.

**Figure 3:**
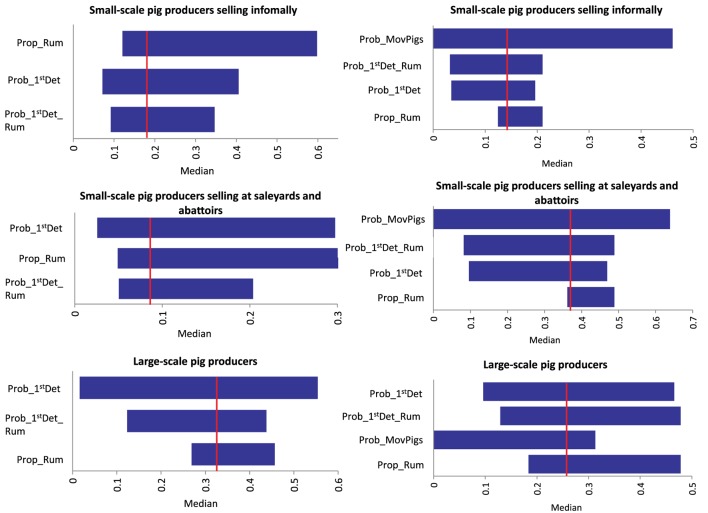
**Outputs of a sensitivity analysis investigating the influence of different input variables to the median (vertical line) probability of two spread scenarios [left: no spread beyond the index farm (*Scenario 1*); right: spread through movement of infective pigs (*Scenario 2*)] of foot-and-mouth disease that has previously been introduced in a piggery in Australia (Sensitivity analysis with 5,000 iterations using @Risk’s Advanced Sensitivity Analysis) (***Prob_1^st^Det_Rum***, Probability of farmer detecting FMD in ruminants; *Prob_1^st^Det*, Probability of farmer detecting FMD in pigs; *Prob_MovPigs*, Probability of movement of pigs during infective period; *Prop_Rum*, Presence of ruminants)**.

**Figure 4:**
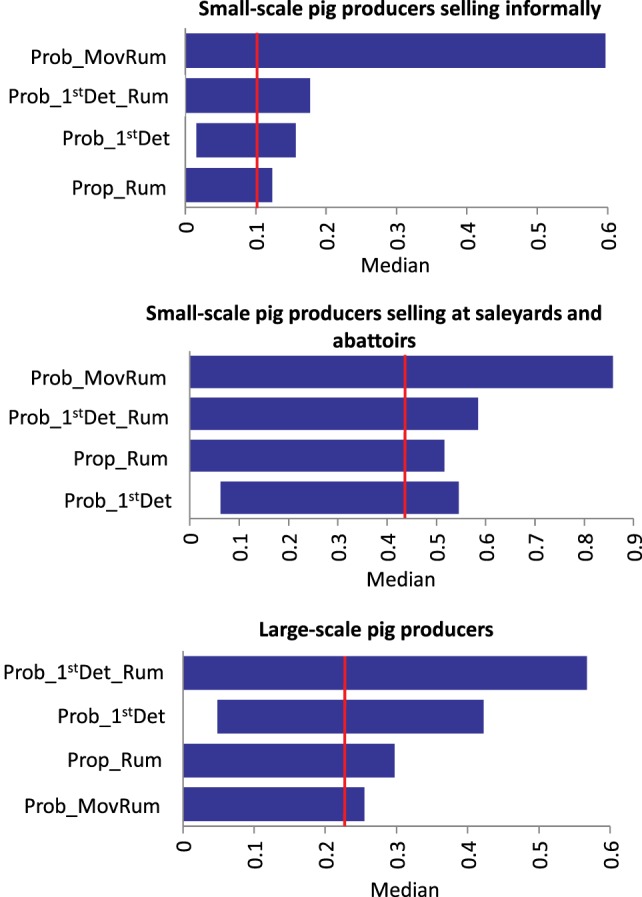
**Influence of different input variables to the median (vertical line) of the scenario of spread through movement of infective ruminants of foot-and-mouth disease that has previously been introduced in a piggery in Australia (Sensitivity analysis with 1,000 iterations using @Risk’s Advanced Sensitivity Analysis) (*Prob_1^st^Det_Rum*, probability of farmer detecting FMD in ruminants; *Prob_1^st^Det*, probability of farmer detecting FMD in pigs; *Prop_Rum*, presence of ruminants; *Prob_MovRum*, probability of movement of ruminants during infective period)**.

Results of the sensitivity analysis on *Scenario 1* and *Scenario 2* for each group of pig producers are presented in Figure 3. For *Scenario 1* (no spread beyond the index farm) among small-scale pig producers, the input parameter most influential on the output of the model is the presence of ruminants on the farm. A significant proportion of these producers stated having ruminants on the farm. If the proportion of producers with ruminants in the piggery is decreased to only 10% (base value 82–85%), the likelihood of *Scenario 1*, increases 35 and 18% in the small-scale producers selling informally and in those selling at saleyards and abattoirs, respectively. For large-scale producers, the probability of the farmer detecting FMD in pigs is the input value with the most influence on the output probability of *Scenario 1*, with 18% increase in the probability of FMD not spreading form the index farm when detection in pigs is 0.9 (base value 0.56). A similar influence of the probability of FMD detection is seen among small-scale producers selling informally. The probability of spread through movement of infective pigs off the farm (*Scenario 2*) decreases when increasing the probability of the farmer detecting in pigs and ruminants for the three groups of pig producers. The influence of these input variables is more significant among large-scale and small-scale pig producers selling at saleyards and abattoirs (decrease of up to 25%). As expected, movement of pigs off farm influences the probability of *Scenario 2* for all piggery types. The probability of spread through movement of contaminated fomites has a significant influence on the occurrence *Scenario 3*, given this model assume that the virus would spread through fomites independently of the farmer detecting the disease. When *Prob_Fomites* is reduced to 0.1 (from a base value of 0.5 for large-scale piggeries and small-scale piggeries selling through saleyards and abattoirs), there is a fivefold decrease in the probability of spread through this pathway. These results stress the importance of maintaining appropriate biosecurity practices to minimize the spread of FMD through fomites, such as pig transport and visitor vehicles, clothes and equipment. For *Scenario 5* (spread through movement of ruminants), the input value with the most influence on the model output is the probability of movement of ruminants during the infective period. However, the influence is different among the three groups of pig producers as seen in Figure 4. The probability of detecting FMD in ruminants has also an important influence on the probability of *Scenario 5* occurring, especially among small-scale piggeries selling through saleyards and abattoirs, which are those with an estimated lower probability of detection (base value 0.24). When detection in these piggeries is improved to 0.89, the probability of *Scenario 5* occurring decreases by 13.5-folds.

## Discussion

Several studies have suggested that small-scale pig producers are more likely to introduce and spread emergency animal diseases (EAD) compared to large-scale pig producers (1, 8, 10, 11, 47, 48), with the lack of appropriate isolation for incoming animals, the use of saleyards for trading pigs, their poor knowledge on EADs and their low compliance with legislative requirements for keeping pigs being the main reasons for this suggestion. The current study is the first comparative assessment on the FMD introduction and spread risks posed by small and large-scale piggeries in Australia, which has used extensive data on producers’ practices. Data used to populate the models in this study is based on several quantitative and qualitative studies among small-scale pig producers in Eastern Australia conducted between 2005 and 2009. These studies, which aimed to investigate biosecurity and surveillance among this sector of the pig industry, have been previously described as providing baseline information on practices and attitudes toward biosecurity among small-scale pig producers in Australia (8). Subsequent studies suggest that biosecurity practices among pig producers have not significantly changed since (48–50). However, although estimates used for the current assessments are considered an accurate representation of practices among pig producers in the country, some practices of producers located in Western areas of the country might not be appropriately represented. The model assumed that FMD was introduced through the illegal importation of 5 kg of cured or salted meat and that the virus survived until exposure occurred. We acknowledge that to accurately estimate the probability of exposure of domestic pigs due to illegal introduction of infected meat, virus survival should be considered; however, the aim of this study was to identify biosecurity practices posing a risk for disease introduction and spread in piggeries and compare this risk between different sectors of the pig industry. These sectors were, small-scale piggeries selling through informal means, small-scale piggeries selling at saleyards and abattoirs and large-scale piggeries. Results of the probabilities of exposure and spread posed by each sector of the industry should be considered relative among the three sectors of the industry.

The probability of exposure of a piggery to the FMD virus, which has been introduced into Australia through the illegal importation of 5 kg of FMD-infected cured or salted meat products was estimated *Extremely low* for the three groups of piggeries. One of the factors driving this low probability is the fact that this assessment considered households without and with pigs having an even probability of illegally introducing meat into the country and most households in Australia do not keep pigs. As such, the probability of the illegally introduced meat being destined to a household with pigs is extremely low. There is no available data providing scientific evidence about households with pigs being more likely to illegally introduce FMD-infected meat into Australia than other households. However, data on illegal movements of meat products are needed to confirm this assumption. The most likely pathway of exposure according to this assessment is through the direct feeding of the infected meat to the pigs. In addition, the sensitivity analysis indicates that exposure is highly influenced the probability of producers to swill feed pigs. Swill feeding was estimated to be more likely among small-scale producers; however, estimates of swill feeding were incorporated in the model with significant uncertainty as they were based on information on feeding practices of producers collected during questionnaires and interviews (8, 9, 12). More accurate information on swill feeding incidence among large and small-scale pig producers in Australia would improve validity of the results. Mathews (51), in a report assessing Australia’s current level of preparedness and capacity to prevent and respond to an outbreak of FMD, identified the effectiveness of swill feeding prohibitions, especially among periurban and small-scale pig producers, as one of the areas that required attention. As previously described, swill feeding has been identified as posing the major risk for FMD introduction and establishment in Australia, through illegally introduced FMD-contaminated meat or dairy products (15, 51). Similarly, the illegal importation of meat, which is subsequently fed to pigs as swill, has been identified as the potential cause of the introduction of emergency diseases, such as CSF and ASF (20, 22, 23). As Schembri et al. (8) indicates, a program involving appropriate swill feeding investigations and effective education and enforcement strategies, supported by a consistent national definition of swill feeding is required to improve current data on swill feeding incidence and producers’ awareness and compliance.

According to results from these assessments, once FMD has been introduced into a piggery, the most likely pathway of spread is through contaminated fomites (*Scenario 3*). Spread of FMD through contaminated vehicles, equipment or clothing with poor or absent appropriate disinfection has been reported as an important pathway of FMD spread from infected properties (15, 52, 53). Spread through this pathway is estimated less likely to occur among small-scale piggeries selling informally due to the low frequency of animal and vehicle movements and the non-use of external staff in these properties compared to other piggeries.

In the current assessment, a similar probability of spread through this pathway was estimated for large- and small-scale piggeries selling at saleyards and abattoirs. Large-scale pig producers have been reported to have better on-farm biosecurity practices in Australia (8, 48) and other countries, such as Finland (47) and United States (54), with these studies suggesting that large-scale producers might perceive the impact of disease as more significant for their enterprise. However, pig movements off farm among large-scale enterprises are frequent and all employ external staff, which could contribute to the spread of the virus through contaminated fomites. By contrast, among small-scale pig producers selling at saleyards and abattoirs pig movements off farm are not as frequent as those among large-scale pig producers. This scenario was considered independent of the farmer detecting the infection, as movement of contaminated fomites could occur before detection of clinical signs occurs, given shedding of the virus could start during the pre-clinical phase of the disease (15, 53). It could be argued that the earlier FMD is detected the less likely the virus would spread through fomites. According to available data, large-scale producers are considered more likely to early detect FMD-infected animals than small-scale producers, and as a consequence, spread through fomites in large-scale piggeries would be less likely to occur. Given this has not been considered in this assessment, the probability of *Scenario 3* to occur in large-scale piggeries could be somewhat overestimated.

The next spread scenario with highest probability among all producers was the spread of FMD through movement of pigs from the index farm (*Scenario 2*). Similarly than for *Scenario 3*, spread through movement of pigs was less likely to occur among small-scale piggeries selling informally due to the lower number of pigs kept on the farm and frequency of pig movements off the farm. However, although the probability of this scenario of spread was lower than for other piggeries, movement of pigs to non-commercial pathways could jeopardize animal traceability in the event of a disease outbreak. Some of these pig properties are not registered within government and/or industry databases, challenging the ability to trace back animal movements in the event of an EAD outbreak and increasing the potential magnitude of the outbreak. A recent study among 198 small-scale pig producers in Australia reported over 85% of participant producers moving pigs off their property in the last 12 months, ~10% of producers not recording animal movements and 3% not having a legally required property identification code for their property (49). Mathews (51) identified the poor understanding on the number and location of small holder producers in Australia and the need for a national register as critical for the management of EADs. For this scenario, the main differences between the three sectors of the pig industry were the different destinations of pigs being moved off the property. While pigs from small-scale piggeries selling informally are mainly moved to other properties keeping pigs, pigs from small-scale piggeries selling at saleyards and abattoirs are mainly sent to saleyards and pigs from large-scale piggeries are sent to abattoirs. This will affect the potential magnitude of the spread of FMD before the outbreak is detected and the potential strategies to reduce the risks of spread. The ability of detecting FMD-infected animals at saleyards and abattoirs, where animals from different origins are commingled, is crucial for limiting the spread of the disease. The estimates of FMD detection used in the current assessments are based on a study by Hernández-Jover et al. (35), who conducted a quantitative evaluation of the likelihood of exotic disease detection with passive disease surveillance activities for pigs at saleyards and abattoirs in eastern Australia. This study indicates that although the probability of detecting FMD at these locations was high when assuming a herd and unit design prevalence of 1 and 30%, respectively, the probability of early detecting FMD at these venues could be improved. This study identified the improvement of disease awareness of saleyard and abattoir stockmen, increased presence of inspectors at these venues and identification of high-risk herds as approaches for enhancing the capacity of the country for early detection of emerging animal diseases. As suggested by several studies, the use of a risk-based surveillance approach, with surveillance being focused at locations with high-risk animals could result in more efficient allocation of resources (55–57).

The sensitivity analysis indicates that for all piggery types, spread of the virus is highly influenced by the probability of the farmer detecting FMD and the presence of ruminants on the farm. No spread beyond the index farm (*Scenario 1*) was more likely to occur in large-scale piggeries as these are considered more likely to detect disease and a lower proportion of these producers keep ruminants (8, 12). Early detection and reporting of FMD is crucial for limiting the spread of the virus and minimizing the potential impact of an outbreak. According to data available, small-scale pig producers have low awareness of EADs and the concept of shared responsibility in relation to the management of EADs. In addition, their contact with veterinarians is low and a lack of trust with government agencies has been identified (8, 49). As a consequence, these data suggest small-scale pig producers would be unlikely to detect FMD before the virus was spread to other livestock in other properties. Similarly, several previous studies identified lack of EAD awareness and negative attitudes toward disease reporting among livestock producers (58–61). Mathews (51) suggested that an incursion of FMD into Australia would not be readily detected, and identified these factors as contributing to the delayed detection of an FMD outbreak. Supporting this suggestion, modeling studies estimated an expected time to FMD detection after being introduced into the country of 22–47 days (53, 56).

## Conclusion

These assessments provide information regarding the relative order of magnitude of the risks of FMD introduction and spread among the three sectors of the pig industry, as well as the biosecurity practices posing higher risks among piggeries for each of the sectors considered. This information can support decision-making when prioritizing resource allocation for improving the capability of the pig industry to prevent and respond against emergency animal disease outbreaks. According to the results of this assessment, direct feeding of the infected meat to the pigs (swill feeding) is the most likely pathway of exposure, and the probability of this to occur is slightly higher among small-scale producers. If FMD is to be introduced into a piggery in Australia, spread is more likely to occur if this piggery is a small-scale piggery selling at saleyards and abattoirs with movement of contaminated fomites and movement of pigs and ruminants off the farm the most likely pathways of spread. Presence of ruminants on the farm and the probability of the farmer detecting FMD are the most influential factors for the spread of the virus. Although large-scale piggeries have higher probability of FMD spread than small-scale piggeries selling informally, they are easy to locate, are members of the pig industry body APL and do not use non-commercial venues to market their pigs. These would limit the potential extent of an outbreak. Small-scale piggeries selling informally do not pose a higher likelihood of spread of disease than the other sectors of the industry; however, if spread from these piggeries occurs, non-traceable movement of pigs would increase the magnitude of an outbreak.

This study suggests that there is a need for improving engagement with biosecurity and animal health management of pig producers and agricultural shows, saleyards and abattoirs stakeholders understanding producers’ current attitudes and behaviors toward biosecurity and animal health management, especially among small-scale producers, and collaboration among government and industry stakeholders are crucial for the development of effective extension strategies that could lead to practice change in relation to biosecurity and EAD management.

## Author Contributions

MH-J led the study, building and implementing the risk assessment models, and writing the manuscript. NS led the data gathering exercises of this study, participated in the estimation of parameter estimates, and providing input in the writing of the manuscript. PH and J-AT participated in the design of the data gathering exercises, provided input in the scenario tree model building and the estimation of the input parameters, and provided input on the writing of the manuscript. PM provided guidance and ongoing input on the building of the risk assessment models, estimation of input parameters and implementation of the models, as well as the writing of the manuscript.

## Conflict of Interest Statement

The authors declare that the research was conducted in the absence of any commercial or financial relationships that could be construed as a potential conflict of interest. The reviewer FS and handling editor declared their shared affiliation, and the handling editor states that the process nevertheless met the standards of a fair and objective review.
