# Supplementary material for: A Comparative Assessment of the Risks of Introduction and Spread of Foot-and-Mouth Disease among Different Pig Sectors in Australia
Source: Front Vet Sci. 2016 Sep 22;3:85. doi: 10.3389/fvets.2016.00085 (PMC5031773; doi:10.3389/fvets.2016.00085)
Supplement: Supplementary file 1 [file Data_Sheet_1.DOCX]

*Online supplementary material*

**Description of scenario trees**

1. **Exposure assessment**

The exposure assessment describes the pathways how a domestic pig in Australia could be exposed to FMD virus from FMD-infected meat being illegally introduced into the country and estimates their corresponding probability to occur. Three exposure assessments were conducted, one for each of the following sectors of the pig industry: 1) small-scale (≤100 sows) piggeries selling at saleyards and abattoirs; 2. Small-scale piggeries selling informally; 3. Large-scale piggeries. The same structure of the scenario tree and nodes are used for the three exposure assessments; however, the input values for some of the nodes differs between three sectors of the pig industry. A detailed description of the nodes and input parameters used for these exposure assessments are described below.

- 1. *Household type*

The household type node had two possible branches, *without pigs* and *with pigs* and estimated the proportion of each type of household (*Prop_withPigs; Prop_withoutPigs*). This node was used as the FMD-infected meat product brought into Australia by an incoming passenger could go to any household in the country. According to the Australian Bureau of Statistics household and family projections from 2011 to 2036 (ABS, 2015), the projected number of households in Australia at 30 of June 2016 will be 9.2 million. Thi*s* projection was based on 2011 Census data. In order to account for uncertainty around this value a Pert distribution with a most likely value of 9.2 million households, a minimum of 8.7 million households (95% of 9.2 million), and a maximum of 9.6 million households (105% of 9.2 million) was used. The number of households with pigs in Australia was estimated from the Australian Pig Annual 2012 -2013 (APL, 2014). According to the estimates provided in this report, in 2012 there were 1,071 breeding pig herds with less than 100 sows, 315 breeding pig herds with more than 100 sows and 524 contract growers. Results of the face-to-face interviews with producers selling through saleyards indicated that 85.6% of small producers were not registered within Australian Pork Limited (APL) (Schembri et al., 2015). In addition, the number of the estimated pig herds in Australia was based on information obtained from the 2011 Census exercise, which only includes a sample of the agricultural business population (ABS, 2015). As a consequence, the estimated number of small scale pig herds is likely to be underestimated. As such, this value was modelled using a Pert distribution with the APL estimate (n = 1,071) as the minimum value, plus 10% and 15% as the most likely and maximum values, respectively. Large-scale producers and contract growers were considered to be registered within APL and the number of these producers was added in the model as fixed values without uncertainty around them. The number of pig herds in the three groups was then added to obtain the total number of households with pigs.

- 1. *Proportion of waste from one serve of meat*

The assessment assumes that 5kg of FMD-infected meat have already been introduced into Australia by incoming passengers. From this amount of FMD-infected meat, only the proportion of meat discarded as waste could be the source of infection of pigs. Thus, the node PROPORTION OF WASTE was included in the model with two possible branches: *Waste* or *No waste*. In order to evaluate the proportion of the meat that will be discarded as waste (*Prop_waste*), the number of serves in 5kg of cured or salted meat was estimated. It was assumed that a single serve of this type meat was in average 50g, ranging from 25 to 100g. These values were added in the model using the Pert distribution (Pert(0.025, 0.05, 0.1)). A survey study in the United Kingdom (Gale, 2002) investigating the proportion of meat discarded as waste, reported that most respondents would discard between 1 to 5% of the meat purchased. Biosecurity Australia (DAFF, 2004) estimated the proportion of pig meat that is discarded by households as waste with a Pert distribution with a minimum of 1%, a most likely of 5% and a maximum of 10%. However, these values were applicable to fresh meat and in the current assessment we are assuming that the imported meat could only be cured or salted products. Accordingly, the research team decided to use lower estimates than those used by Biosecurity Australia. For this assessment, the proportion of cured or salted meat discarded by households as waste was estimated with a Pert distribution with a minimum of 1%, a most likely of 2% and a maximum of 5%.

- 1. *Piggery type*

The piggery type node was a category node with two branches, *Large piggery* and *Small piggery*. Large-scale piggeries included breeding herds with more than 100 sows and also contract growers. The input values used for this node have been previously explained in 1.1. and the corresponding proportions were calculated (*Prop_Small*; *Prop_Large*).

- 1. *Small-scale piggery type*

This node (SMALL-SCALE PIGGERY TYPE) was added to distinguish between small-piggeries selling through saleyards and abattoirs and those selling mainly through informal means. The two branches of the node were *Selling informally* and *Selling through saleyards and abattoirs*. The proportion of these two types of piggeries (*Prop_Informal*; *Prop_Sal&Ab*) among small-scale piggeries was estimated from data obtained during different studies used as data sources for this assessment. The estimated number of small-scale piggeries in Australia has been explained in 1.1. Among the total of small-scale producers participating in these studies (n = 589), only 37 did mainly sell their pigs through informal means. As most of these studies were focused on producers selling through saleyards, the proportion of producers selling mainly informally was considered to be lower than the actual proportion among all small-scale producers. To add uncertainty around this value a Beta distribution was used as the minimum value of a Pert distribution, and 50 and 70% was added to this minimum to obtain the most likely and the maximum values.

- 1. *Access of feral pigs to waste*

The infected meat discarded as waste could get into contact with feral pigs. It was considered that the probability of the infected waste being accessible to and in contact with feral pigs would depend on the origin of the waste and different estimates for this probability were used for waste originated from piggeries and for that originated from households without pigs. The rationale for this difference was that feral pigs were assumed more likely to be around piggeries than around households without pigs and that some piggeries might discharge the household waste in their property. As a consequence, two nodes were included in the model: 1. ACCESS OF FERAL PIGS_1, probability of waste from households without pigs getting in contact with feral pigs (*Prob_Access1*); and, 2. ACCESS OF FERAL PIGS_2, probability of waste from piggeries getting in contact with feral pigs (*Prob_Access2*). The branches for these nodes were *Yes* or *No*.

The probability of waste from households without pigs getting in contact with feral pigs (*Prob_Access1*) was estimated from the Biosecurity Australia IRA (DAFF, 2004). This assessment considered the probability of the waste being accessible to and located by the feral pigs, and estimated different probabilities depending on the location of the household in Australia. The probability of the waste being accessible was high in remote areas, moderate in rural areas and very low at large towns. The probability of the waste being located by the feral pigs was estimated as *Very low* in remote areas, *Extremely low* in rural areas and *Negligible* at large towns. From these qualitative values, we obtained quantitative values using a uniform distribution following the semi-quantitative methodology described at the Guidelines for Import Risk Analysis (DAFF, 2004), and the probabilities of waste being accessible to and located by feral pigs were multiplied to obtain the probability of waste getting in contact with feral pigs (*Very low* in remote areas, *Extremely low* in rural areas and *Negligible* at large towns). Moreover, as this assessment did not consider the location of households, a combined quantitative estimate for the three regions was calculated using the proportion of households (3% remote, 11% rural and 86% large towns) and the probabilities of waste being in contact with feral pigs in each of these regions, in a cumulative distribution.

The probability of waste from piggeries getting in contact with feral pigs (*Prob_Access2*) was assumed to be higher than that from households without pigs, considering that some piggeries might discharge household waste in their property. As data was not available, the estimate used was based on the value for the probability of waste from households without pigs being in contact with feral pigs. This value was included as the minimum value of a Pert distribution, adding 15% and 20% for the most likely and maximum values, respectively. Large-scale piggeries were assumed not to discharge household waste in their property and as a consequence (*Prob_Access2*) was not used in the assessment for this sector of the pig industry.

- 1. *Infection of feral pigs*

The probability of the feral pigs being infected once they are in contact with the FMD-contaminated waste (*Prob_Infection*) will depend on the dose of the virus in the meat product and the viability of the virus. The node INFECTION OF FERAL PIGS had to possible outcomes, *Infection* or *No infection*. Estimates used for this input were sourced from the Biosecurity Australia IRA (DAFF, 2004) and the Australian Veterinary Emergency Plan (AUSVETPLAN) for FMD (Animal Health Australia, 2014). The probability that the infected meat will contain sufficient dose to cause infection of feral pigs was estimated as *High*. Tissues of infected animals contain high concentration of virus and pigs are easily infected by the oral route. Frequently, past FMD outbreaks were originated in pigs ingesting contaminated meat products (Bourn, 2002; Nunn 2001). According to the 1994 USDA: APHIS:VS paper ‘*Foot and Mouth Disease - Sources of Outbreaks and Hazard Categorization of Modes of Virus Transmission*’, of 627 known sources of FMD outbreaks in the world from 1870 to 1993, 66% of these were caused by infected meat or meat products (USDA:APHIS:VS, 1994). Viability of the virus in the infected waste until the feral pig will get in contact with this waste was also estimate as *High*. Although the virus is inactivated by desiccation and heat, it could survive for a long period of time in the environment. Previous research reported a virus survival time of 50 days in water, 74 days on pasture (8 to 18^o^C and high humidity), 26 to 200 days in soil, hay or straw, 35 days on cardboard, wood or metal contaminated with blood or tissue and 398 days on wood contaminated with fat (Animal Health Australia, 2014). The FMD virus is inactivated in 3 days in meat after a normal post-slaughter acidification; however if the ph is above 6.2, as in rapidly chilled meat, the virus can survive for longer time (Cottral 1960 in Animal Health Australia, 2014). Moreover, the virus can survive in chilled or frozen lymph nodes, bone marrow, viscera and residual blood clots, and salted and cured meats for months.

These qualitative estimates were translated into quantitative values using a uniform distribution as previously described and the probabilities of sufficient dose and viability were multiplied to obtain the probability of feral pigs being infected by the FMD-contaminated waste.

- 1. *Contact of feral pigs with domestic pigs*

For this scenario, domestic pigs are those pigs from the index piggery, which will be a different type of piggery according to the three assessments. Contact between feral pigs and domestic pigs can occur depending on the type of housing of the pigs and the presence of feral pigs around the piggery. As explained in the previous sections, feral pigs could have been infected through ingestion of waste from any type of household (without pigs, large-scale piggeries, small-scale piggeries selling through saleyards and abattoirs, and small-scale piggeries selling informally). The probability that feral pigs infected via waste originated in the index piggery will contact pigs from the same piggery was considered higher than the probability that feral pigs infected via waste from other households will contact pigs from the index piggery, as the feral pigs would first need to travel to this piggery. Thus, two nodes were used for estimating these values: 1. CONTACT OF FERAL PIGS_1, probability that feral pigs infected via waste from other households will contact pigs from the index piggery (*Prob_Contact1*); and, 2. CONTACT OF FERAL PIGS_2, probability that feral pigs infected via waste originated in the index piggery will contact pigs from the same piggery (*Prob_Contact2*). The branches of these nodes were *Yes* or *No*.

To estimate the (*Prob_Contact2*), data collated during the data gathering exercises and literature were used. Information used for this estimate was in relation to the presence of feral pigs close to the piggery, the presence of a fence around the property and the type of housing. The input values used for the three types of piggeries considered in this assessment were estimated as follows:

- *Small-scale piggery selling through saleyards and abattoirs:* According to the studies conducted for this assessment (Schembri et al., 2010b; Schembri et al., 2015), among 109 producers selling at saleyards and abattoirs, 37 of them reported seeing feral pigs close to the property and 53% of those had their pigs housed outdoors. Pearson et al. (Pearson et al., 2014; Pearson et al., 2016) reported that among 62 small-scale producers, 8 of them had seen feral pigs in their property and 6 of these had free-range operations. The probability that feral pigs infected via waste originated from a small-scale piggery selling at saleyards and abattoirs, will contact pigs from the same piggery (*Prob_Contact2*) was estimated as the proportion of producers reporting feral pigs around their property as a high proportion of these housed their pigs outdoors, using a Beta distribution (Beta(46, 127)).
- *Small-scale piggery selling informally:* Of 21 producers selling informally participating in the data gathering exercises (Schembri et al., 2010b; Schembri et al., 2015), 5 of them reported seeing feral pigs close to their property and 4 of them had their pigs housed outdoors. Although 4 of them also had a perimeter fence it was considered that a fence would not stop FMD transmission between animals. From these values, the probability that feral pigs infected via waste originated from a small-scale piggery selling through informal means, will contact pigs from the same piggery (*Prob_Contact2*) was estimated based on the presence of feral pigs close to the property and uncertainty was added using a Beta distribution around this proportion (Beta(6, 17)).
- *Large-scale piggery:* Results of the data gathering studies described in the manuscript, indicate that of the 41 large-scale producers with information regarding feral pigs, 11 reported seeing these animals close to their property and of these, 3 housed their pigs outdoor (Schembri et al., 2015). Pearson et al. (Pearson et al., 2014; Pearson et al., 2016) reported that among 108 large-scale producers, 12 had seen feral pigs; however, all of these producers had intensive piggeries, with animals being housed indoors. As housing of the pigs was considered an important factor when estimating the probability of contact between feral and domestic pigs, this probability was estimated with a Pert distribution, using the proportion of producers seeing feral pigs (23/149) as the maximum value, minus 20% and 50% as the most likely and minimum values, respectively.

The *Prob_Contact1* was estimated using a Pert distribution with *Prob_Contact2*, minus 25% and 50%, as maximum, most likely and minimum, respectively, for the three types of piggeries.

- 1. *Swill feeding*

The SWILL FEEDING node had to possible outcomes, *Swill feeding* or *No swill feeding*. The probability of swill feeding (*Prob_Swill*) among producers was estimated from findings of the studies used as data sources for this assessment (Schembri et al., 2010a). Feeding practices that could pose a risk for swill feeding and the lack of understanding of swill feeding were used to estimate the potential probability of these producers feeding swill to their pigs. These feeding practices included feeding meat, table scraps or retail waste from bakeries or supermarkets to the pigs. The input values used for this node different among the three types of piggeries as described below.

- *Small-scale piggery selling through saleyards and abattoirs:* This input value was estimated based on the feeding practices of small-scale producers selling at saleyards and abattoirs participating in the study by Schembri et al (2010a). Of 109 producers of this exposure group, 19 reported feeding practices that could pose a risk for swill feeding. As the proportion of producers reporting practices that could pose a risk for swill feeding might be overestimating the actual proportion of producers swill feeding, high uncertainty was used around this value. The input value was included in the model using a Pert distribution with this proportion of producers (19/109) as the most likely value, and minus 50% and plus 20%, as the minimum and maximum values of the distribution, respectively.
- *Small-scale piggery selling informally:* Among 22 producers with information on these topics, 5 reported feeding practices that could pose a risk for swill feeding (Schembri et al., 2010a; Schembri et al., 2010b). Similarly than for the small-scale producers selling through saleyards and abattoirs, this probability was included in the model with a Pert distribution, using the proportion of producers with potential risky feeding practices (5 of 22 producers), minus 50% and plus 20%, as the most likely, minimum and maximum values, respectively.
- *Large-scale piggery:* Among 41 large-scale producers with information on feeding practices, 6 reported feeding practices considered to pose a risk for swill feeding (Schembri et al., 2015). The probability of large-scale producers to swill feed was incorporated into the model with a Pert distribution, using the proportion of producers practicing potential risky feeding practices (6/41), minus 50% and plus 20%, as the most likely, minimum and maximum values, respectively.

1. **Consequence assessment**

The partial consequence assessment investigated the potential outbreak scenarios after the first pig from a piggery (small- or large-scale) is exposed to the FMD virus. Two consequence scenario trees were developed to represent the potential spread of the FMD virus from small and large-scale piggeries. The only difference in the structure between these two scenarios was the potential destinations where pigs from the index farm could go once moved off the farm. A detailed description of the nodes and input parameters used for both consequence assessments follows.

- 1. *Clinical signs*

The CLINICAL SIGNS node is included in the scenario as only animals displaying clinical signs could be detected by the farmer of the first exposed piggery or in successive destinations. The two branches of this node are *Yes* or *No*. Once the first piggery is exposed, spread of the infection could occur before the infected animals show clinical signs, as the farmer would not be able to detect the infected animals. In this case, infection could spread through movement of animals, contaminated fomites and people carrying the virus. The probability that an infected animal would display clinical signs (*Prob_CS*) will depend on the incubation period as well as the number of days pigs are infective. The incubation period of FMD in pigs could vary from 1 to 9 days approximately (Alexandersen and Donaldson, 2002; DAFF, 2004; Animal Health Australia, 2014) and was incorporated in the model using a lognormal distribution truncated to a minimum of 1 day and a maximum of 12 days. Animals can shed FMD virus for up to four days before the onset of symptoms, and many studies have found that pigs cleared the infection within 3 to 4 weeks. Accordingly, the infective period in pigs could go from approximately 14 days (7 days incubation period and 7 days of clinical signs) to 30 day, and was modelled using a uniform distribution using these values as minimum and a maximum infective period. The probability of a random animal in the infected farm showing clinical signs was then calculated ((Infective period – Incubation period) / Infective period). However, when animals are not showing clinical signs before they are moved from the index farm, the model considers the probability of showing clinical signs at the next destination of the animals (*Prob_CS_2*), and the estimated value is higher than *Prob_CS*. The time elapsed from infection to movement of animals (considered up to 2 days) was subtracted from the incubation period. The obtained time (time to the onset of clinical signs) was then used to calculate the probability of a random animal in the first destination showing clinical signs ((Infective period – Time to the onset of clinical signs) / Infective period), which was higher than *Prob_CS*.

- 1. *Farmer detection and reporting in pigs*

Following the previous node, if the infected animal was showing clinical signs, the next node in the scenario was FARMER DETECTION IN PIGS AND REPORTING, which estimates the probability of the farmer from the first exposed piggery detecting and reporting the FMD infection (*Prob_1^st^Det*). The two outcomes for this node were *Detection* or *No detection*. For this input value, two components were estimated separately, the probability of the farmer detecting and the probability of the farmer reporting the FMD case or suspect to the authorities. Information from the studies conducted to collect data for this assessment (*see Data sources of manuscript*), as well as the previous studies investigating biosecurity and disease reporting among pig producers (Schembri et al., 2006; Schembri et al., 2010b; Schembri et al., 2015), was used for the estimation of these probabilities. The probability of farmer detecting a FMD-infected animal was estimated from questions related to disease recognition (pictures with pigs with signs of FMD, erysipelas and ringworm provided), health records kept and training on exotic animal diseases (EAD). The input values used for the three types of piggeries considered in this assessment are described below:

- *Small-scale piggery selling through saleyards and abattoirs:* Information used in this node was available from 89 producers selling at saleyards and abattoirs. Of these, 98.9%, 79.8% and 69.7% recognized FMD, erysipelas and ringworm, respectively. Over half of producers kept health records and only 5% of producers had some EAD training. When considering also findings of Schembri et al. (2006) among producers selling at saleyards, the probability of the farmer detecting a FMD case was estimated with a Pert distribution with 0.4 as the most likely value with an uncertainty of 20% around this value. This estimation is lower than that used for the small-scale piggeries selling informally (RiskPert(0.4, 0.5, 0.6)) as data suggests that the motivation for the small-scale producers selling at saleyards and abattoirs to keep pigs is mainly to generate an economic income, being less likely to keep pigs as pets and as a consequence less concerned about the health of their pigs. Among these producers, only 16.8% used a veterinarian in the previous 12 months, 96.6% would report signs of FMD, 20% previously reported unusual diseases and 86% would follow the correct action in the event of an unusual disease. This information supported by findings of the focus groups regarding barriers to disease reporting (Schembri et al., 2015), suggest that the probability of these producers to report would be lower than that of the small-scale producers selling informally. Thus, the probability of the farmer reporting in this assessment was estimated using a Pert distribution with 0.6 as the most likely value and an uncertainty of 20% around this value. The overall *Prob_1^st^Det* was obtained multiplying the probability of the farmer detecting (RiskPert (0.32, 0.4, 0.48)) and the probability of the farmer reporting (RiskPert (0.48, 0.6, 0.72)).
- *Small-scale piggery selling informally*: Among producers selling informally, all were able to recognize FMD disease, and 80% and 73.3% recognized erysipelas and ringworm, respectively. Half of producers kept some type of health records and only 16.7% of producers stated having had some EAD training. According to Schembri et al. (2006), 25% of all vendors at the saleyards and approximately 50% of small-scale vendors had an exotic disease knowledge rating of 2 or less of 5. Most were unable to describe the signs of a vesicular disease and there was a general lack of awareness about the risks of swill feeding. Although we consider that the small-scale producers selling informally are likely to be concerned about the health of their animals as some raise rare breeds, are organic pig producers or keep pigs as pets, from the data collected, the probability of the farmer detecting a FMD case was estimated using a Pert distribution with a most likely value of 0.5, and with a minimum and maximum of less and plus 20% of the most likely value, respectively. For the estimation of the probability of the farmer reporting the FMD case once it is detected, questions regarding the use of a veterinarian, the reporting of a FMD case (pictures with pigs with signs of FMD provided), previous reporting incidences, and actions followed in the event of an unusual disease, were used. Among producers selling informally, 18.9% used a veterinarian in the previous 12 months, 100% would report an animal with visible FMD clinical signs, 33.3% previously reported unusual diseases and 100% would follow the correct action in the event of an unusual disease. However, focus groups participants stated the following barriers to disease reporting (Schembri et al., 2015): Fear of negative consequences, such as quarantine, prosecution, negative reputation, loss of stock and livelihood; overreaction from the media and the government; lack of compensation; and, previous negative experiences. Although all producers knew who they had to contact in case of an unusual disease and all would report a FMD outbreak, due to the negative comments about reporting during the focus group discussions and the low proportion of producers using a veterinarian, the probability of the farmer reporting was modelled using a Pert distribution with 0.75 as the most likely value, and less and plus 20% as the minimum and maximum values, respectively. The overall *Prob_1^st^Det* was then obtained multiplying the probability of the farmer detecting (RiskPert (0.4, 0.5, 0.6)) and the probability of the farmer reporting (RiskPert(0.6, 0.75, 0.9)).
- *Large*-*scale piggery:* Among large-scale producers participating in the data gathering exercises conducted for this assessment, 93%, 86% and 79% recognized FMD, erysipelas and ring worm, respectively. A higher proportion (86%) of these producers compared to the small-scale ones kept health records of the pigs and had some training on EAD recognition (22%). From this findings and considering that the larger the producer is the more likely that the piggery will have a pig veterinarian or consultant, adequate training for EAD recognition, the probability of the farmer detecting a FMD-infected animals was estimated using a Pert distribution with a most likely value of 0.7 and a range of +/- 20%. Moreover, large-scale producers are more likely to keep pigs as a primary source of income, being more concerned about the profitability of the piggery and as a consequence about the health of their pigs. A high proportion of large-scale producers (86%) used a veterinarian in the previous 12 months, 93% would report signs of FMD, 33% previously reported some incidence on the farm and all of them would do the correct action in the event of an unusual health event. These findings suggest that the probability of a large-scale producer reporting a FMD suspect outbreak would be higher than that of a small-scale producer, and was estimated with a Pert distribution with a most likely value of 0.8 and +/- 20% as the maximum and minimum values, respectively. The probability of the farmer detecting (RiskPert(0.56, 0.70, 0.84)) and the probability of the farmer reporting (RiskPert(0.64, 0.80, 0.96)) were multiplied to obtain the overall *Prob_1^st^Det*.
  1. *Presence of ruminants on the farm*

The extent of the spread of FMD from the index farm will be dependent, among other factors, on the presence of ruminants on the same farm where pigs are kept. A high proportion of pig farms have ruminants on their property. Infected pigs excrete high quantity of virus on their breath and cattle are very susceptible to airborne infection, requiring less virus to get infected than pigs by the respiratory route (Donaldson and Alexandersen, 2001, 2002). If the index farm has ruminants on the same property where pigs are housed, and the farmer does not detect the disease in very early stages, it is very likely that ruminants will become infected. This node estimates the proportion of pig farms keeping also ruminants (*Prop_Rum*). The PRESENCE OF RUMINANTS node had two possible branches, *Yes* or *No*. The input values used for the three types of piggeries follows:

- *Small-scale piggeries selling through saleyards and abattoirs:* Most small-scale producers (460 of 552) selling through saleyards and abattoirs stated having ruminants on the farm (Schembri et al., 2015). This proportion was incorporated into the model using a Beta distribution to add uncertainty around the values (RiskBeta(461,93)).
- *Small-scale piggeries selling informally:* Most small-scale producers (32 of 37) selling informally stated having ruminants on the farm (Schembri et al., 2010b). This proportion was incorporated into the model using a Beta distribution to add uncertainty around the values (RiskBeta(33,6)).
- *Large-scale piggeries:* Over half of large-scale producers (64 of 87) participating in previous studies stated having ruminants on the farm (Schembri et al., 2015). This proportion was incorporated into the model using a Beta distribution to add uncertainty around the values (RiskBeta(65,24)).
  1. *Farmer detection and reporting in ruminants*

If ruminants are present in the index piggery and the farmer does not detect FMD in pigs, the virus would most likely spread to ruminants. The farmer could then detect the infection in these species. The symptoms and severity of FMD vary with the species of animal, and the serotype and strain of the virus. Cattle are often the first to show signs of the disease, and as such are usually called *Disease indicators*. However, sheep and goats show only mild signs, acting as *Maintenance hosts* (Animal Health Australia, 2014). From the branch *Yes* of the PRESENCE OF RUMINANTS node, the next node in the scenario was FARMER DETECTION AND REPORTING IN RUMINANTS, which estimates the probability of the farmer from the first exposed piggery detecting and reporting the FMD infection in ruminants (*Prob_1^st^Det_Rum*). The two outcomes for this node were *Detection* or *No detection*. No specific information on the farmer ability to recognize FMD in ruminants was collected during the previous studies and this probability was based on the estimated probability of detection in pigs and the presence of cattle on the farm (as they are *Disease indicators*). The input values used for the three types of piggeries are described below:

- *Small-scale piggeries selling through saleyards and abattoirs:* This probability was based on the presence of cattle (Disease indicators) on the farm and also on the previously estimated probability of the farmer detecting FMD in pigs (RiskPert (0.32, 0.4, 0.48)). If cattle are infected, farmers would be more likely to detect FMD in this species than in pigs. Previous studies indicated that approximately 85% of those small-scale pig producers selling formally and keeping ruminants on the farm have cattle. The probability of detecting FMD in ruminants was estimated higher than the probability of detection in pigs. The probability of these producers detecting FMD in pigs was used as the minimum value of a Pert distribution, and 10 and 20% were added to it to obtain the most likely and maximum values. Probability of reporting was considered the same (RiskPert(0.48, 0.6, 0.72)) than that estimated when the farmer detected FMD in pigs.
- *Small-scale piggeries selling informally:* This probability was based on the presence of cattle on the farm, as this species are more likely to show clinical signs than sheep and goat, and also on the previously estimated probability of the farmer detecting FMD in pigs (RiskPert (0.4, 0.5, 0.6)). It is expected that if cattle are infected, farmers would be more likely to detect FMD in this species than in pigs, as according to literature, cattle are considered disease indicators for FMD. Previous studies indicated that approximately 82% of those small-scale pig producers selling informally and keeping ruminants on the farm have cattle. Accordingly, the probability of detecting FMD in ruminants if the disease was not previously detected in pigs was estimated higher than the probability of detection in pigs. For this, the probability of these producers detecting FMD in pigs was used as the minimum value of a Pert distribution, and 10 and 20% were added to it to obtain the most likely and maximum values. Probability of reporting was considered the same (RiskPert(0.6, 0.75, 0.9)) than that estimated when the farmer detected FMD in pigs.
- *Large-scale piggeries:* Farmer detection and reporting in ruminants: As for the previous groups, this probability was based on the presence of cattle (Disease indicators) on the farm and also on the previously estimated probability of the farmer detecting FMD in pigs (RiskPert (0.56, 0.7, 0.84)). Previous studies indicated that approximately 75% of those large-scale pig producers and keeping ruminants on the farm have cattle. The probability of detecting FMD in ruminants was estimated higher than the probability of detection in pigs. The probability of these producers detecting FMD in pigs was used as the minimum value of a Pert distribution, and 10 and 20% were added to it to obtain the most likely and maximum values. Probability of reporting was considered the same (RiskPert(0.64, 0.8, 0.96)) than that estimated when the farmer detected FMD in pigs.
  1. *Probability of pig movement during infective period*

If the farmer of the first exposed piggery does not detect the FMD-infected animal/s, for the spread to occur through animal movement, animals must move off the farm during the infective period. The PIG MOVEMENT DURING INFECTIVE PERIOD node estimated the probability of pig movements during the infective period (*Prob_MovPigs*) and had two possible branches, *Yes* or *No*. Pigs excrete virus from 1 day before showing clinical signs and during 4 to 5 days. Pigs do not become carriers of the FMD virus. Information on frequency of movements off the farm was collected during the face-to-face interviews with producers (Schembri et al., 2015) and the case study interviews (Schembri et al., 2010b). It was considered that weekly to monthly movements would allow for a potential spread of the disease. Input values used for the three types of piggeries differed as described below:

- *Small-scale piggeries selling through saleyards and abattoirs:* Frequency of movements from small-scale piggeries selling at saleyards and abattoirs is higher than that from small-scale piggeries selling informally. Considering weekly to monthly movements as those which would allow for movement of infective animals from the index farm, 60.6% of small-scale producers selling at saleyards and abattoirs would potentially move animals during this infective period. Thus, the probability of animal movement during the infective period was estimated with a Pert distribution with a most likely value of 0.6 and an uncertainty of 20% around this value.
- *Small-scale piggeries selling informally:* Findings from the studies gathering data for this assessment, suggest that movements from piggeries selling by informal means are not very frequent as only one third of producers were selling weekly to monthly. The probability of pig movement during the infective period was estimated with a Pert distribution with a most likely value of 0.3, and +/- 20% as the maximum and minimum values to account for uncertainty around the estimate.
- *Large-scale piggeries:* The probability of animal movement during the infective period depends on the frequency of movement of pigs off the farm. All large-scale producers participating in the data gathering exercises reported moving animals in a weekly to monthly frequency, most of them moving animals off the farm every week. This suggests that large-scale producers are very likely to move animals during the infective period, and the probability of this movement to occur was estimated using a Pert distribution with a maximum of 1 and minus 5 ad 10% as the most likely and minimum values, respectively.
  1. *Probability of ruminant movement during infective period*

Moreover, if the index farm has ruminants on the same property, it is very likely that ruminants will become infected and spread the infection. Cattle can become carriers of the virus and potentially infect other susceptible animals during the latent stage of the infection (Salt, 1993). However, if the farmer detect the infection in pigs it is assumed that infected ruminants will also be detected, and movement of these animals off farm will not occur. The RUMINANT MOVEMENT DURING INFECTIVE PERIOD node estimated the probability of ruminant movement during the infective period (*Prob_MovRum*) and had two possible branches, *Yes* or *No*. Information on the movement of ruminants off the farm, such as potential destinations and frequency of movements, was not collected during this assessment and a qualitative estimation based on literature and depending on the type of farm and the number of ruminants on the property has been used. The input values used for the three types of piggeries follows:

- *Small-scale piggeries selling through saleyards and abattoirs:* A qualitative estimate based on the number of ruminants kept on the farm was used to estimate this probability. Some animals carry FMD virus for prolonged periods after recovering from acute disease. FMD virus can persist for up to nine months in sheep and up to four months in goats, while most cattle carry this virus for six months or less. However, some animals remain persistently infected for up to 3.5 years being able to transmit the virus to other animals if they come in close contact (Animal Health Australia, 2014). As a consequence, if ruminants become infected and disease is not detected, it is very likely that movement of ruminants off the farm would cause the spread of the disease. Those small-scale pig producers selling formally and keeping ruminants on the farm, keep a significant number of sheep (n = 762) and cattle (n = 93) on average. Although, approximately 40% of these producers had less than 30 heads of sheep or cattle, a significant proportion have more than 1000 sheep (46%) or 100 cattle (30%). When comparing these values with those presented for small-scale piggeries selling informally it seems that the probability of ruminant movement during the infective period would be higher in this group evaluated here. Accordingly, this probability was estimated *moderate.*
- *Small-scale piggeries selling informally:* Information on the frequency of movement of ruminant off the piggery was not collected and as a consequence a qualitative estimate based on the number of ruminants kept on the farm was used. Small-scale pig producers selling informally keep a lower average number of ruminants (sheep and cattle) than the other two types of piggeries. Most of these producers (>55%) keep less than 30 heads of sheep or cattle, with less than 10% with more than 1000 sheep or 100 cattle. Accordingly, the probability of ruminant movement during the infective period was estimated *low* for this group of producers.
- *Large-scale piggeries:* A qualitative estimate based on the number of ruminants kept on the farm was used to estimate this probability. Those large-scale pig producers keeping ruminants on the farm, kept an average of 1,925 sheep and 224 cattle. Similarly than for the previous group, a significant proportion of these large-scale producers kept more than 1000 sheep (36%) or 100 cattle (42%). As these producers keep more ruminants on the farm than small-scale piggeries, the probability of ruminant movement during the infective period for large-scale producers was estimated *high*.
  1. *Pig movement from the index farm*

Movement of infected animals could occur if the animals are not showing clinical signs or if the farmer from the first exposed piggery does not detect and report the FMD outbreak. This category node (PIG MOVEMENT FROM THE INDEX FARM) was added as pigs moving off the farm can have several possible destinations and estimated the proportion of movement of pigs to each of these destinations. These destination differed according to the type of piggery. Six possible destinations (branches of the node) where pigs from a small-scale piggery could be moved to, were considered: 1. *Small-scale piggery (Prop_SCpiggery)*; 2. Person who keeps pigs as pets (*Private individual; Prob_PrivateInd)*); 3. *Agricultural show (Prop_AS)*; 4. Property for home-kill (*Home-kill; Prop_HK)*); 5. *Saleyard (Prop_Saleyard)*; and, 6. *Abattoir (Prop_Abattoir)*. For large-scale piggeries, four destinations were considered: 1. Large-scale piggery (*Prop_LSpiggery*); 2. *Small-scale piggery (Prop_SCpiggery); 3. Saleyard (Prop_Saleyard)*; and, 4. *Abattoir (Prop_Abattoir)* According to the destination, different potential outbreak scenarios can occur. This node accounted for the proportion of movements of animals from a small-scale piggery to each of the six potential destinations described and data was sourced from the studies conducted to collect information for this assessment (*see Data sources of manuscript*). Animals from the index farm can move to different destinations, thus to estimate the proportion of movements to each destination, the total number of movements stated by producers was considered. These proportions were included in the model using Beta distributions. Inputs used for the three types of piggeries are detailed below:

- *Small-scale piggeries selling through saleyards and abattoirs:* There were a total of 883 movements stated by 552 small-scale producers selling at saleyards and abattoirs. Among these movements, 113 were to another small-scale piggery, 26 to a private individual, 5 to agricultural shows, 192 to a property for home consumption, 455 to saleyards and 82 to abattoirs. Beta distributions around the proportion of movements to each destination were used to incorporate these values into the model.
- *Small-scale piggeries selling informally:* According to the data collected, among 57 movements stated by producers, 32 were to another small-farm, 10 to a private individual, 10 to a property for home-kill, 4 to an agricultural show and 1 to a domestic abattoir. No movements to saleyards were reported by participant producers; however, a Beta distribution was also added as movement to this destination could potentially happen.
- *Large-scale piggeries:* To estimate the proportion of movements to the different destinations from a large-scale piggery, information from the studies conducted in this assessment and from a report on the structure of the pig industry in Australia (Cutler and Holyoake, 2007) was used. According to the current studies, among 114 movements reported by 87 large-scale producers, 23% were to other farms, 36% to saleyards and 50% to abattoirs. Nine large-scale pig producers were interviewed in the Cutler and Holyoake (2007) study. Among 14 movements reported by these producers, 9 were to abattoirs, 2 to saleyards and 3 to other farms. Considering this information, an estimate of 65% was used for abattoir movements, 15% for saleyard movements, 10% for large-scale farms and 10% for small-scale farms. Pert distributions were used to estimate the proportions for each destination, using the estimate as the most likely value and adding +/-20% around this estimate.
  1. *Ruminant movement from the index farm*

As previously stated, information on potential destinations of ruminants from the index farm and the frequency of these movements was not collected during this assessment as one of the main objectives of this study was to evaluate to risk of informal and formal pig movements. This category node (RUMINANT MOVEMENT FROM THE INDEX FARM) was included in the model to acknowledge this potential spread scenario, but was not investigated in detail. According to literature (AusVet Animal Health Services, 2005, 2006; Hassall & Associates Pty Ltd, 2006), the most likely potential branches for this node would be: 1. *Saleyard (Prop_SaleyardRum)*; 2. *Abattoir (Prop_AbattoirRum)*; 3. *Contractor (Prop_Contractor)*; 4. *Independent property (Prop_IndProp)*; and, 5. *Export (Prop_Export)*. The likelihood and impact of the spread scenarios as a consequence of ruminant movement from the index farm was qualitatively discussed. It was considered that if infected ruminants move off the farm, spread in different extent would occur.

- 1. *Detection at a large-scale piggery*

If the infected pig was not showing clinical signs at the index farm (large-scale piggery) or was not detected and reported by the farmer, the animal could be moved to another large-scale-scale piggery. This node (DETECTION AT A LARGE-SCALE PIGGERY) only applies to the large-scale piggery scenario tree. The node estimated the probability that the farmer at the large-scale piggery receiving the infected pigs would detect infection (*Prob_LSDet*) and had to possible outcomes, *Detection* or *No detection*. To estimate *Prob_LSDet*, the probability of the farmer detecting and the probability of the farmer reporting the FMD outbreak were evaluated. The same data sources and input estimates than those used for the *Prob_1^st^Det* for large-scale piggeries were used for the estimation of the input values of this node (*see 2.2*.).

- 1. *Detection at a small-scale piggery*

If the infected pig was not showing clinical signs at the index farm or was not detected and reported by the farmer, the animal could be moved to another small-scale piggery. For this node (DETECTION AT A SMALL-SCALE PIGGERY) no distinction was made between small-scale piggeries selling informally and those selling through saleyards and abattoirs. The node estimated the probability that the farmer at the small-scale piggery receiving the infected pigs would detect infection (*Prob_SSDet*) and had to possible outcomes, *Detection* or *No detection*. To estimate *Prob_SSDet*, the probability of the farmer detecting and the probability of the farmer reporting the FMD outbreak were evaluated. The same data sources than those used for the *Prob_1^st^Det* were used for the estimation of the input values of this node; however, data from those small-scale piggeries buying pigs from other small-scale piggeries was considered. Information related to quarantine measures applied for the introduction of new stock, the use of boots and overalls only for the farm, disease recognition, health records and training on EAD, was used to estimate the probability of the farmer detecting (Schembri et al., 2010b; Schembri et al., 2015). According to data collected, among small-scale producers considered in this node, none had EAD training, half of them kept health records and 75% reported that FMD was a serious disease. Moreover, only 8% applied correct quarantine measures for new stock and 35% had boots and overalls for on-farm use, indicating a low probability of detection when the infected animal is introduced to this small-scale piggery. This is supported by Schembri et al. (2006) findings among producers selling at saleyards, which have been previously described. As a consequence, the probability of a farmer in small-scale piggery detecting FMD was estimated with a Pert distribution with a most likely value of 0.4, and with a minimum and maximum values of less and plus 20% of the most likely value, respectively. The probability of the farmer from the small-scale piggery reporting was calculated similarly than the probability of reporting at the index piggery, using information on the use of a veterinarian, the reporting of a FMD case, previous reporting incidences, and actions followed in the event of an unusual disease. Almost all producers would report FMD (98%) and would follow the correct action in the event of an unusual disease (91.7%); however, only 17.3% of these producers used a veterinarian in the previous 12 months and 20% previously reported unusual diseases. Moreover, as previously explained, focus group participants stated important to disease reporting. As a consequence, the probability of the farmer reporting was modelled using a Pert distribution with 0.6 as the most likely value, and less and plus 20% as the minimum and maximum values, respectively. Multiplying the probability of the detection (RiskPert (0.32, 0.4, 0.48)) and reporting (RiskPert (0.48, 0.6, 0.72)), the overall *Prob_SSDet* was obtained.

- 1. *Detection at a private individual*

This node represents the probability of detection by a person who bought the animal from the small-scale piggery to be kept as a pet (*Prob_PrivateIndDet*). For this detection node (DETECTION AT A PRIVATE INDIVIDUAL) data collected from small-scale producers who bought animals to keep as pets or from those who stated hobby as the main motivation for keeping pigs was considered (Schembri et al., 2010b; Schembri et al., 2015). The two outcomes of the node were *Detection* or *No Detection*. Data sources and information used are the same than those used for estimating the *Prob_SSDet*. Among these producers, all of them would identify FMD clinical signs and 55% kept health records; however, none had training on EAD, only 11% apply correct quarantine measures for new stock and 36% use boots and overalls on-farm. According to these findings, the probability of detection was estimated using a Pert distribution with a most likely value of 0.4, with +/- 20% as the maximum and minimum values. Information related to the probability of reporting, indicates that almost all producers would report FMD (92.1%) and would follow the correct action in the event of an unusual disease (86.7%), but only 17% had used a veterinarian in the previous year. Considering these findings, the probability of the private individual to report was estimated with the same values than those used for the *Prob_SSDet*, with the Pert distribution RiskPert (0.48, 0.6, 0.72).

- 1. *Detection at an agricultural show*

The DETECTION AT AN AGRICULTURAL SHOW node had two branches, *Detection* or *No detection*. The probability of detection at agricultural shows (*Prob_ASDet*) was estimated from data collected in a previous study evaluating biosecurity practices at agricultural shows in Australia (Cha et al., 2009). Detection of a FMD outbreak at this location is based on detection of clinical signs of FMD in infected animals. According to this study, only 11.7% of agricultural shows required a health status declaration for incoming pigs based on clinical signs or laboratory tests. Among the 59 agricultural shows visited, monitoring the health of the pigs at the agricultural shows was responsibility of the exhibitors, the agricultural show staff or the veterinarian. The study reported that only 3.3% of the agricultural show staff had training in disease recognition. For this assessment, probability of detection was assumed to be depending on the person responsible for the health of the pigs. Shows were categorized in three groups: shows where health was responsibility of the exhibitors or pig producers only (28.3%), shows where the agricultural show staff was involved with the health monitoring (42.4%) and shows where veterinarians were involved with this activity (28.3%). The probability of exhibitors or pig producers detecting a FMD case was considered the same than the *Prob_1^st^Det*, which has been previously described. A previous study evaluating the post-farm-gate surveillance system for the pig industry in Australia (Hernández-Jover et al., 2011), estimated that the probability of the saleyard stockmen to detect a FMD-infected animal was low and used the Pert distribution RiskPert(0.1, 0.3, 0.5) for this estimation. The ability of the agricultural show staff to detect a FMD-infected animal was considered to be similar than the probability of the stockmen detection at saleyards and the same estimate was used. The assessment considered that if the animal was showing FMD clinical signs, veterinarians would be able to detect them, and as a consequence, detection of veterinarians was added to the model as a fixed value of 1. As an overall estimation of the probability of detection was needed for all type of agricultural shows, the previous estimates were combined considering the relative proportions among the different types of shows. Uncertainty around these proportions was added using Beta distributions.

- 1. *Detection at a property for home-kill*

Infected animals from the index farm could be sold or moved to another property with the purpose of home consumption and the node DETECTION AT A HOME-KILL with the two branches *Detection* or *No detection*, was included in the model. To estimate the probability of detection of a FMD-infected animal in this type of properties (*Prob_HKDet*), data from small-scale pig producers who kept animals for home consumption was used, and the probability of detection and reporting were estimated separately. Data sources are the same that those used for estimating the *Prob_SSDet* (Schembri et al., 2010b; Schembri et al., 2015). Approximately 90% of these producers would identify FMD clinical signs, 60% recorded health information and 35% had boots and overalls for on-farm use only. However, only 3% applied the correct quarantine measures for new stock and none of the producers had training on EAD. As these producers were keeping pigs mainly for home consumption, and according to the data collected, the probability of detection of a FMD-infected animal was estimated with a Pert distribution with a most likely value of 0.4, with a range of 20% as the minimum and maximum values (RiskPert(0.32, 0.40, 0.48)). According to the data, approximately 77% of producers would report FMD, most of them (95%) would follow the correct action in the event of an unusual disease, approximately 40% of producers used a veterinarian in the last 12 months and none of them had previously reported any incidence. Considering also the barriers to reporting identified at the focus group discussions (Schembri et al., 2015), the probability of reporting of producers who kept pigs for home-kill was estimated with a Pert distribution with a most likely value of 0.6, with a range of 20% for the minimum and maximum values (RiskPert(0.48, 0.60, 0.72)).

- 1. *Detection at a saleyard*

The DETECTION AT SALEYARDS node had to possible outcomes, *Detection* or *No detection*. The probability of detection at saleyards (*Prob_SaleyardDet*) has been previously investigated in a study evaluating the post-farm-gate disease surveillance for emerging animal diseases at swine saleyards and abattoirs in Australia (Hernández-Jover et al., 2011). This study considered two different types of saleyards according to the destination of the animals sold at this location: Domestic saleyards, those saleyards supplying only domestic buyers (producers, butchers and domestic abattoirs); and, export saleyards, those saleyards supplying domestic and export buyers (export abattoirs). According to this study, the probability of detection (median, 5 and 95 percentiles) at domestic and export saleyards was 0.475 (0.343, 0.599) and 0.474 (0.334, 0.603). This probability represents the ability of detecting a FMD-infected animal showing clinical signs. As the current study did not differentiate between saleyard types, the estimation of the *Prob_SaleyardDet* was obtained combining the probability of detection at the domestic and export saleyards according to the proportion of each type of saleyard in Australia. A cumulative distribution with the 5 percentiles of the output distributions of the probability of detection at domestic and export saleyard obtained in the previous study (Hernández-Jover et al., 2011) was used for this estimation. The output of the two cumulative distributions was then multiplied by the proportion of domestic (76.9%) and export saleyards (23.1%) in Australia (Cutler and Holyoake, 2007) and both values added to obtain a single estimate.

- 1. *Detection at abattoirs*

The DETECTION AT ABATTOIRS node had to possible outcomes, *Detection* or *No detection*. The probability of detection at pig domestic and export abattoirs in Australia (*Prob_AbattoirDet*) was also evaluated at the abovementioned study by Hernández-Jover et al. (2011). According to this study, the probability of detecting an infected animal, at the domestic abattoir (median, 5 and 95 percentiles) was 0.43 (0.329, 0.534) and that at the export abattoir was 0.861 (0.799, 0.916). The probability of detection at each abattoir was then estimated using a cumulative distribution with the 5 percentiles of the output distribution of the probability of detection at each type of abattoirs (Hernández-Jover et al., 2011). As for the saleyards, the output of the two cumulative distributions was then multiplied by the proportion of domestic (73.1%) and export abattoirs (26.9%) in Australia (William Salter, personal communication) and both values added to obtain a single estimate.

- 1. *Movement of contaminated fomites from the index farm*

Foot and mouth disease is most likely to be spread from the index farm by the movement of infected animals; however, transmission of the virus could also occur by mechanical means or fomites, such as via animal contact with virus on the surfaces of transportation vehicles, other equipment or clothing (Alexandersen et al., 2003). The virus could be spread to another farm, saleyards or abattoirs through contaminated fomites. For this reason, the MOVEMENT OF FOMITES FROM THE INDEX FARM node was included in the model to estimate the probability of transmission of FMD through fomites moving from the index farm (*Prob_Fomites*) and had two possible outcomes, *Yes* or *No*. Fomites could be contaminated footwear, veterinary equipment, clothing, farm equipment, bedding, and transportation vehicles. Fomites can become contaminated from direct contact with the mouth or feet of infected animals or from contact with the virus shed onto the ground. Although the virus is rapidly inactivated by heat, low humidity and change in pH, depending on the climate conditions, the virus can survive in clothing and footwear up to 9 weeks, in walls up to 2 weeks, in hay up to 4 weeks and in soil up to 20 weeks (Animal Health Australia, 2014). Cleaning and disinfecting fomites would most likely inactivate the virus. As Orsel et al. (2009) indicated, transmission of the virus could occur before infected animals show clinical signs, and the period of time between the introduction of the virus and the detection is considered a high risk period for transmission. Thus, although the farmer could detect the FMD-infected animal, transmission through contaminated fomites could occur during the high risk period of transmission, when the animal is not showing clinical signs. In pigs, this period is only considered to be 1 day, as pigs excrete virus from 1 day before showing clinical signs and during 4 to 5 days.

As a consequence, this assessment assumed that spread through contaminated fomites was independent of farmer detection and other factors were considered for the estimation of this probability. Data sources for this estimation of the probability of transmission through contaminated fomites (*Prob_Fomites*) where those used for the estimation of the *Prob_SSDet* (Schembri et al., 2010b; Schembri et al., 2015). The factors considered were practices of producers, such as the frequency of pig movements off the farm, using specific boots and/or overalls in the piggery, using their own truck or vehicle to transport the pigs, cleaning the truck after each use and allowing visitors to the farm. The input values used in each of the piggery types are described below:

- *Small-scale piggeries selling through saleyards and abattoirs:* Among producers selling at saleyards and abattoirs, only 35% had boots and/or overalls for on-farm use only, almost all of them used their own vehicle to transport pigs off the farm, 80% cleaned the vehicle after each use and 70% allowed visitors in the piggery. Movement of pigs off the farm was more frequent than in small-scale producers selling informally, with approximately 60% of producers moving pigs off farm in a weekly to monthly basis. According to these results, the probability of transmission of FMD through contaminated fomites was considered to be *Moderate*. As previously described, the quantitative values for the analysis were obtained using a uniform distribution following the semi-quantitative methodology of the Guidelines for Import Risk Analysis (DAFF, 2004).
- *Small-scale piggeries selling informally:* Among these producers, approximately 80% stated using boots and/or overalls specific for the farm, all of them used their own truck or vehicle to transport pigs, 83% cleaned the vehicle after each use and 50% allowed visitors in the piggery. As previously stated movement of animals from small-scale piggeries selling informally and as a consequence of potential contaminated vehicles was not very frequent. Moreover, these piggeries do not employ staff to work with the pigs, reducing the probability of contamination of clothing. According to these findings, the probability of transmission of FMD through fomites was considered to be *Low*. The quantitative values for the analysis were obtained using a uniform distribution following the semi-quantitative methodology of the Guidelines for Import Risk Analysis (DAFF, 2004).
- *Large-scale piggeries:* Data from large-scale pig producers indicate that 66% of producers have boots and/or overalls for on-farm use only, approximately 50% use an external transporter to transport their pigs off the farm, 85% usually clean their truck after each use and 78% allowed visitors into their property. Moreover, as previously explained, movement of animals off the farm was very frequent, with most producers moving animals every week, and they are more likely to employ external staff than small-scale producers. However, biosecurity practices of these producers were considered better than those applied by small-scale producers. As a consequence, the probability of transmission of FMD through contaminated fomites was considered to be similar than that used for small-scale producers selling at saleyards and abattoirs, and was estimated as *Moderate*. Quantitative values were obtained using a uniform distribution as previously explained (DAFF, 2004).
  1. *Movement of contaminated people from the index farm*

Another potential pathway of spread of the FMD virus from the index farm could be through movement of contaminated people. Although the most likely way of people carrying the virus would be in contaminated clothing, which would be considered as a fomite transmission, humans could also carry the virus in their upper respiratory tract for up to 28 hours (Kitching et al., 2005; Morgan, 2006; Animal Health Australia, 2014). The MOVEMENT OF CONTAMINATED PEOPLE FROM THE INDEX FARM node was included in the model, with two possible outcomes, *Yes* or *No*. However, transmission through people carrying the virus is considered unlikely to occur. To estimate the probability of transmission through movement of people carrying the virus in their respiratory tract (*Prob_People*), practices of producers in relation to washing their hands after handling pigs, allowing visitors into the farm, having a controlled entry of visitors and working in another piggery, were considered. Input values used differed according to the piggery type as follows:

- *Small-scale piggeries selling through saleyards and abattoirs:* Approximately 60% of small-scale producers selling at saleyards and abattoirs stated washing their hands after handling their pigs and had controlled entry for visitors. Moreover, 70% allowed visitors to the farm. One of these producers reported working in another piggery. However, these producers are not likely to employ staff to work on the piggery. According to these findings, this assessment considered the probability of transmission through people carrying the virus in their respiratory tract as *Low*. Quantitative values were obtained using a uniform distribution as previously explained.
- *Small*-*scale piggeries selling informally:* Movement of contaminated people from the index farm: All producers stated washing their hands after handling their pigs, 73% of producers had controlled entry of visitors to the farm and 50% allowed visitors to the farm. None of the producers worked in another piggery. Although visitors might be allowed to the piggery it is unlikely that they will have direct contact with the pigs. Thus, this assessment considered the *Prob_People* as *Very low*. As for the previous node, the quantitative values for the analysis were obtained using a uniform distribution following the semi-quantitative methodology of the Guidelines for Import Risk Analysis (DAFF, 2004).
- *Large-scale piggeries:* The input value used for large-scale producers was the same than that used for small-scale producers selling at saleyards and abattoirs. Over half of large-scale producers stated washing their hands after handling their pigs and although 78% allowed visitors into the producers, 80% had a controlled entry of visitors. None of these producers reported working in another piggery; however, it is considered likely that large-scale produces employ external staff. The probability of transmission through people carrying the virus in their respiratory tract was estimated as *Low*. Quantitative values were obtained using a uniform distribution as previously explained.

**References**

ABS, 2015. 3236.0 - Household and Family Projections, Australia, 2011 to 2036. In: Statistics, A.B.o. (Ed.).

Alexandersen, S., Donaldson, A.I., 2002. Further studies to quantify the dose of natural aerosols of foot-and-mouth disease virus for pigs. Epidemiology and Infection 128, 313-323.

Alexandersen, S., Quan, M., Murphy, C., Knight, J., Zhang, Z., 2003. Studies of Quantitative Parameters of Virus Excretion and Transmission in Pigs and Cattle Experimentally Infected with Foot-and-Mouth Disease Virus. Journal of Comparative Pathology 129, 268-282.

Animal Health Australia, 2014. Disease strategy: Foot-and-mouth disease (Version 3.4). Australian Veterinary Emergency Plan (AUSVETPLAN). In: Council, A.H.A.P.I.M. (Ed.), Canberra, ACT.

APL, 2014. Australian Pig Annual 2012-2013. In: Limited, A.P. (Ed.), 88.

AusVet Animal Health Services, 2005. A Review of the Structure and Dynamics of the Australian Dairy Cattle Industry. In: Australian Government, D.o.A.F.a.F. (Ed.), 79.

AusVet Animal Health Services, 2006. A Review f the Structure and Dynamics of the Australian Beef Cattle Industry. In: Australian Government, D.o.A.F.a.F. (Ed.), 93.

Cha, E., Toribio, J.A.L.M.L., Thomson, P.C., Holyoake, P.K., 2009. Biosecurity practices and the potential for exhibited pigs to consume swill at agricultural shows in Australia. Preventive Veterinary Medicine 91, 122-129.

Cutler, R.S., Holyoake, P.K., 2007. The structure and dynamics of the pig meat industry. In: Australian Government, D.o.A.F.a.F. (Ed.).

DAFF, 2004. Generic Import Risk Analysis (IRA) for Pig Meat: Final Import Risk Analysis Report. Biosecurity Australia, Canberra.

Donaldson, A.I., Alexandersen, S., 2001. Relative resistance of pigs to infection by natural aerosols of FMD virus. Vet Rec 148, 600.

Donaldson, A.I., Alexandersen, S., 2002. Predicting the spread of foot and mouth disease by airborne virus. Revue Scientifique Et Technique De L Office International Des Epizooties 21, 569-575.

Gale, P., 2002. Risk Assessment: Use of composting and biogas treatment to dispose of catering waste containing meat. Department for Environment, Food and Rural Affairs,,, 183.

Hassall & Associates Pty Ltd, 2006. The structure and dynamics of Australia’s sheep population. In: Australian Government, D.o.A.F.a.F. (Ed.).

Hernández-Jover, M., Cogger, N., Martin, P.A.J., Schembri, N., Holyoake, P.K., Toribio, J.A.L.M.L., 2011. Evaluation of post-farm-gate passive surveillance in swine for the detection of foot and mouth disease in Australia. Preventive Veterinary Medicine 100, 171-186.

Kitching, R.P., Hutber, A.M., Thrusfield, M.V., 2005. A review of foot-and-mouth disease with special consideration for the clinical and epidemiological factors relevant to predictive modelling of the disease. The Veterinary Journal 169, 197-209.

Morgan, I.R., 2006. Spread of Foot-and-Mouth Disease: A Review prepared for the Australian Meat Research Corporation. In: Science, V.I.o.A. (Ed.).

Orsel, K., Bouma, A., Dekker, A., Stegeman, J.A., Jong, d.M.C.M., 2009. Foot and mouth disease virus transmission during the incubation period of the disease in piglets, lambs, calves, and dairy cows. Preventive Veterinary Medicine 88, 158-163.

Pearson, H.E., Toribio, J.-A.L.M.L., Lapidge, S.J., Hernández-Jover, M., 2016. Evaluating the risk of pathogen transmission from wild animals to domestic pigs in Australia. Preventive veterinary medicine 123, 39.

Pearson, H.E., Toribio, J.A.L.M.L., Hernandez-Jover, M., Marshall, D., Lapidge, S.J., 2014. Pathogen presence in feral pigs and their movement around two commercial piggeries in Queensland, Australia. Veterinary Record 174, 325.

Salt, J.S., 1993. The carrier state in foot and mouth disease—an immunological review. British Veterinary Journal 149, 207-223.

Schembri, N., Hart, K., Petersen, R., Whittington, R., 2006. Assessment of the management practices facilitating the establishment and spread of exotic diseases of pigs in the Sydney region. Australian Veterinary Journal 84, 341-348.

Schembri, N., Hernandez-Jover, M., Toribio, J.A., Holyoake, P.K., 2015. On-farm characteristics and biosecurity protocols for small-scale swine producers in eastern Australia. Prev Vet Med 118, 104-116.

Schembri, N., Hernández-Jover, M., Toribio, J.A., Holyoake, P.K., 2010a. Feeding of prohibited substances (swill) to pigs in Australia. Australian Veterinary Journal 88, 294-300.

Schembri, N., Holyoake, P.K., Hernández-Jover, M., Toribio, J.L.M.L., 2010b. A qualitative study of the management and biosecurity practices of 13 interviewed pig owners selling via informal means in New South Wales, Australia. Animal Production Science 50, 852-862.
